# Sex-specific influences of mtDNA mitotype and diet on mitochondrial functions and physiological traits in *Drosophila melanogaster*

**DOI:** 10.1371/journal.pone.0187554

**Published:** 2017-11-22

**Authors:** Wen C. Aw, Michael R. Garvin, Richard G. Melvin, J. William O. Ballard

**Affiliations:** 1 School of Biotechnology and Biomolecular Sciences, University of New South Wales, New South Wales, Sydney, Australia; 2 School of Biological Sciences, Washington State University, Pullman, Washington, United States of America; University of Arkansas, UNITED STATES

## Abstract

Here we determine the sex-specific influence of mtDNA type (mitotype) and diet on mitochondrial functions and physiology in two *Drosophila melanogaster* lines. In many species, males and females differ in aspects of their energy production. These sex-specific influences may be caused by differences in evolutionary history and physiological functions. We predicted the influence of mtDNA mutations should be stronger in males than females as a result of the organelle’s maternal mode of inheritance in the majority of metazoans. In contrast, we predicted the influence of diet would be greater in females due to higher metabolic flexibility. We included four diets that differed in their protein: carbohydrate (P:C) ratios as they are the two-major energy-yielding macronutrients in the fly diet. We assayed four mitochondrial function traits (Complex I oxidative phosphorylation, reactive oxygen species production, superoxide dismutase activity, and mtDNA copy number) and four physiological traits (fecundity, longevity, lipid content, and starvation resistance). Traits were assayed at 11 d and 25 d of age. Consistent with predictions we observe that the mitotype influenced males more than females supporting the hypothesis of a sex-specific selective sieve in the mitochondrial genome caused by the maternal inheritance of mitochondria. Also, consistent with predictions, we found that the diet influenced females more than males.

## Introduction

Sex-specific differences in energy metabolism have motivated research into their evolution and their underlying mechanisms. Sex-specific evolutionary differences are predicted to occur because it is only females that transmit the cellular energy-producing mitochondria, in the majority of species [1]. Mechanistically, one clear difference lies in the production and survival of gametes, the sperm, and the egg. Males produce numerous, small, and highly mobile sperm that are disposable. In contrast, females produce a small number of large and immobile eggs [2]. While the male shares autosomal and sex-chromosomes, the female provides not only these chromosomes but also cytosolic energy and nutrients for the embryo to develop. As a consequence, mechanistic sex-specific differences in gamete production are predicted. Thus, from the beginning of reproduction, major sex-specific differences are present and likely occur at multiple levels. In this study, we aim to identify evolutionary and mechanistic sex-specific effects on mitochondrial functions and related physiological traits in two *Drosophila melanogaster* lines.

It has been convincingly argued from an evolutionary perspective that the uniparental inheritance of mtDNA can result in sexually antagonistic selection that can cause significant organelle and physiological fitness differences between males and females [1, 3]. With low rates of paternal leakage and recombination, it has been hypothesized that deleterious maternally inherited mtDNA mutations in females will be eliminated by natural selection whereas mutations that harm only males may get passed on [1, 4, 5] (but see Wade and Brandvain [6]). These processes have been termed the Mother’s Curse [5]. A prediction from the Mothers Curse hypothesis is that mtDNA mutations may be deleterious in males but not females. An example is that a mtDNA hypomorph of cytochrome c oxidase subunit II impairs male fertility without affecting other male or female traits including lifespan, heat intolerance, and mechanical shock [3]. In this study, we include males and females from two *Drosophila* lines that have known differences in their mtDNA harbored in a standardized nuclear genetic background.

Based on the mechanistic metabolic flexibility hypothesis [7], we predicted females would exhibit greater metabolic plasticity than males. Energy and substrates that drive mitochondrial respiration can be obtained from glycolysis, beta-oxidation of fatty acids or oxidation of amino acids. Most cells can shift dynamically between these processes in response to the dietary changes, and this is known as “metabolic flexibility” [7]. Females are predicted to have higher metabolic flexibility than males due to their greater reproductive needs and greater insulin sensitivity, which is mediated by sex-specific hormone profiles likely including juvenile hormone in insects [8, 9] and estrogen in humans [10, 11]. These differences in metabolic flexibility may be expressed at the level of phenotype. For example, the energetic requirements for reproduction are hypothesized to have driven the evolution of differences in body size, aggression and sociability in many mammals and insects [12, 13].

To test the metabolic flexibility hypothesis, we fed two *Drosophila* lines four foods that differed in their protein: carbohydrate (1:2, 1:4, 1:8 and 1:16 P:C) ratios. We predicted the metabolic flexibility hypothesis would be supported if females respond more to dietary change than males. Protein and carbohydrate are the two major energy-yielding macronutrients in the fly diet, and their ratio has been shown to have profound impacts on mitochondrial function through alternating the production of mitochondrial metabolites and influence various aspects of physiology, behavior, and life history traits [14–16]. The tested ratios span the normal physiological range encountered in nature. Jensen and colleagues [17] investigated the sex-specific effects of protein and carbohydrate in *D*. *melanogaster* that originated from the Dahomey fly stock. They observed that lifespan was maximized at a high intake of carbohydrate and a low intake of protein in both sexes, whereas nutrient intake had divergent effects on reproduction. Male reproduction was maximized on a high carbohydrate diet, whereas female egg production was maximized on a high protein diet.

Four mitochondrial function traits were assayed in this study. These traits included mitochondrial Complex I oxidative phosphorylation (CI-OXPHOS), mtDNA copy number, reactive oxygen species (ROS) production and superoxide dismutase (SOD) activity [18]. CI-OXPHOS was assayed because we have previously shown a dietary difference in Complex I respiration in males of the same fly lines [15]. MtDNA copy number has been shown to increase with cellular demand and was quantified in this study [19]. ROS are traditionally viewed as toxic and involved in the accumulation of cellular damage that leads to aging. However, accumulating evidence indicates that ROS are also important signaling molecules that regulates cellular functions [20–22]. Cellular SOD can reduce the intracellular concentration of ROS, and total SOD activity was measured. Oxidative stress only occurs when cellular ROS levels exceed a threshold that can be overcome by the cellular antioxidant activity [23].

We assayed four physiological traits. These included fecundity, survival, lipid content and starvation resistance. Female fecundity is highly energy demanding [24] and sensitive toward dietary changes [25]. Studies have demonstrated that a reduction in female fecundity tends to accompany an extension of lifespan in a wide variety of organisms including humans [26], mice [27], *Drosophila* [25, 28, 29] and seed beetles [30]. Lipid content was included because a metabolic switch toward glycolysis can induce accumulation of acetyl-CoA and enhance lipid formation [31, 32]. Starvation resistance was included as this trait is positively correlated with lipid proportion in *Drosophila* [33].

We included *Drosophila* fly lines that harbor distinct mtDNA types (Alstonville and Japan) in a constant nuclear background [34]. The lines have one non-synonymous, two tRNA and four rRNA differences [34, 35]. The non-synonymous ND2 gene mutation in Complex I of the Japan line has been predicted to be functionally important [36], and we structurally model it based on the known *Escherichia coli* structure [37, 38]. When fed a ~1:3 P:C diet male Japan flies harboring the mutation have previously been shown to exhibit increased mtDNA copy number and rates of ROS production as well as decreased male fertility, lipid proportion, starvation resistance and survival [34, 35, 39]. The effects of this mutation on females were unknown.

Our results suggest sexual dimorphism in mitochondrial functions and physiological traits. Consistent with the predictions of the Mother Curse hypothesis, we found the nonsynonymous mutation in Japan affected males more severely than females [1, 5]. Also, we show that female physiological traits are more susceptible to dietary changes than male traits and we argue this is an effect of greater metabolic flexibility in females than in males [10].

## Materials and methods

### Fly lines

The flies were originally collected from Alstonville, Australia, and from Jume, Japan [40]. Fly lines harboring each mtDNA type in the standard *w*^*1118*^ nuclear genome were initially constructed by chromosome replacement using balancers and differed only in their mitochondrial genomes and chromosome 4 [40]. To summarize, chromosomes X, 2, and 3 from the wild-type lines were replaced with homozygous chromosomes from the *w*^*1118*^ *iso* line. To ensure that slightly deleterious mutations had not accumulated before the study, both lines were backcrossed to the standard *w*^*1118*^ for six generations before the study. We have now backcrossed these lines for more than 20 generations suggesting any variation in chromosome 4 is now minimal. We refer to these lines as (*Mitochondria; Nuclear*): *Alst; w*^*1118*^ and *Jap; w*^*1118*^.

Mito-nuclear interactions have been shown to mediate mitochondrial OXPHOS and metabolic function [41]. To test for mito-nuclear interactions, we introgressed *Alst; w*^*1118*^ and *Jap; w*^*1118*^ into an Oregon R genetic background using balancer chromosomes. The *w*^1118^ nuclear genome diverged from the wild caught Oregon R line in 1984, and they have been separated for at least 800 generations. Our introgression protocol followed Zhu and colleagues [42], with the slight modification that we replaced FM6 with FM7. We then backcrossed these lines to Oregon R for five generations to introgress chromosome 4, immediately before experimentation. Hereafter we refer to these lines as *Alst; OreR* and *Jap; OreR*.

### Mitotype differences

The protein coding, tRNA and rRNA regions of Alstonville and Japan mtDNA have been independently sequenced [35, 40, 43]. Based on mitochondrial function studies, the non-synonymous His182Tyr ND2 variant in the electron transport chain Complex I of the Japan line was predicted to be functionally important [15]. However, we cannot definitively state all other mutations are strictly neutral.

To identify the location of the amino acid sites in ND2 we first created an alignment of all available sequences. We included the sequences for the subunits of Complex I from *E*. *coli* because this was used to generate the highest resolution three-dimensional structure [37, 38]. The sequences for 11 *Drosophila* lines that were not part of this work were included to determine if sites were fixed or segregating within *D*. *melanogaster*. The initial alignment was performed with CLC Bio Sequence Viewer 7 (Qiagen Bioinformatics). Small manual adjustments were made based on residues that were highly conserved among diverse taxa as done previously [44]. We then identified homologous residues on the 3-dimensional structure (PDB 3RKO) with Visual Molecular Dynamics software [45]. The R-groups and their orientation were then taken into account in combination with the functional model that has been proposed for proton translocation.

Here we sequenced the A+T rich region because Salminen and colleagues [46] have shown that the non-coding A+T rich region in mtDNA can alter mitochondrial biochemistry and life history traits. Mitochondria were extracted and mtDNA isolated using a DNeasy Kit (Qiagen). Sequencing was performed using Pacific Biosciences (PacBio) RSII Chemistry P6-C4, 10kb template preparation and sequencing with 3.24 μg input DNA at the Ramaciotti Center at University of New South Wales. Sequences were assembled using FALCON 1.8.2 (Pacific Biosciences of California, Inc.) and aligned and annotated to the *D*. *melanogaster* A + T rich region [47, 48] using Geneious 9.1.8 (Biomatters Ltd.).

### Fly maintenance and assay times

Flies were maintained at 23± 0.5° C, 50± 2% relative humidity, 12: 12 hr light: dark cycle and maintained on instant fly food (Carolina Biological Supply). The density of flies in bottles was strictly controlled for two generations before each study and during all experiments. Flies were genotyped at the beginning and end of each study to verify the correct lines were included [35]. Lines did not harbor *Wolbachia* infection or p-elements.

To provide insight into the process of senescence, mitochondrial functions and physiological traits (except survival) were assayed at 11 d and 25 d of age. *D*. *melanogaster* is in the first third of its lifespan at 11 d [40, 49]. The latter age was chosen to represent “old” flies. In the laboratory, the lifespan of *Drosophila* flies is dependent on temperature, diet, genotype and assay method [50]. In field cages, *D*. *melanogaster* has been shown to live for approximately 110 days in the antipodal winter [51].

### Study diets

Flies were fed four different P:C diets (1:2 P:C, 1:4 P:C, 1:8 P:C and 1:16 P:C), all with 680 calories/ L. The food was made by varying sucrose and autolyzed yeast with a standard base containing 1% agar, 0.1% nipagen, 0.1% propionic acid and 0.01% phosphoric acid. The final concentration of the food was set as 180 g• L^-1^. The autolyzed yeast (MP Biomedicals), contains 45% protein, 24% carbohydrate, 21% indigestible fibre, 8% water and 2% acids, minerals and vitamins. We posit that any differences in micronutrients between the diets had a minimal effect on our results, though this needs to be tested. Jensen and colleagues [17] constructed 29 artificial diets and report a similar dietary induced trade-off to that observed in this study.

For each study, eggs were collected by placing oviposition resources (solidified agar-based medium containing 4% agar and 10% molasses molded in large Petri dishes) in cages. The eggs were washed off the oviposition resources with 3% bleach, and water [52] and about 160 eggs (20 μL) added to instant fly food (Carolina Biological Supply). When flies eclosed, 10 males and 10 females of each genotype were placed in 30-mL vials containing one of the four different P:C diets. All flies were transferred into new vials containing fresh food every 3 d unless otherwise stated.

### Mitochondrial function traits

#### CI-OXPHOS

CI-OXPHOS of male and female flies was assayed because the *Jap; w*^*1118*^ line harbors a putative His182Tyr substitution in ND2 [40]. Following Pichaud and colleagues [53], two thoraces were permeabilized in a petri dish with 2.0 mL of relaxing solution BIOPS (2.77 mM CaK_2_EGTA, 7.23 mM K_2_EGTA, 5.77 mM Na_2_ATP, 6.56 mM MgCl_2_, 20 mM Taurine, 15 mM Na_2_Phosphocreatine, 20 mM Imidazole, 0.5 mM Dithiothreitol, 50 mM K-MES, pH 7.1) complemented with 63 μg• mL^-1^ Saponin and gently mixed at 4°C for 30 min. Fibre bundles were then rinsed at 4°C for 10 min in respiration medium (120 mM KCl, 5 mM KH_2_PO_4_, 3 mM Hepes, 1 mM EGTA, 1 mM MgCl_2_ (hexahydrate) and 0.2% BSA (w/ v), pH 7.2.) and transferred into a Oxygraph-2k respirometer (Oroboros Instruments). Before measuring the respiration, oxygen was injected into the chamber to allow maintenance of hyper-oxygenated levels (~550 nmol• mL^-1^) to eliminate any oxygen diffusion limitation.

The respiratory assay was performed at 23°C, and the respiration medium solubility was calculated using the method of Rasmussen [54]. The substrate-uncoupler-inhibitor protocol was: pyruvate + malate + L-proline (each at 10 mM to achieve state II respiration for Complex I, CI Leak), ADP (5 mM to achieve state III respiration for Complex I, CI-OXPHOS), cytochrome c (10 μM, as an indicator of functional integrity of mitochondrial outer membrane, CIc-OXPHOS) and rotenone (0.5 μM, residual oxygen consumption after inhibition of Complex I). The data were analyzed with DatLab (Oroboros Instruments) and expressed as mean respiration rates in pmol O^2^• s^-1^• mg^-1^ fibre± SEM corrected with residual oxygen consumption after inhibition of Complex I (sample size *n* = 6 replicate/ line/ sex/ age/ diet).

To test the generality of the results obtained with *Alst; w*^*1118*^ and *Jap; w*^*1118*^ flies we examined CI-OXPHOS of *Alst; OreR* and *Jap; OreR* flies fed the 1:4 P:C diet. We selected the 1:4 P:C diet as it was an intermediate food and was broadly representative of all diets. We observed that the primary difference occurred between mitotypes and the influence of the nuclear genetic background was not significant. As a consequence, we focused on the *w*^*1118*^ nuclear genetic background in subsequent studies.

#### MtDNA copy number

In *Drosophila*, mtDNA copy number is higher in females than males [55] and may be influenced by mitotype [56]. Here, copy number was measured from total DNA extracted from one fly thorax using the Gentra Puregene Cell Kit (Qiagen). Quantitative PCR (qPCR) was performed in triplicate using a Rotorgene-3000 (Corbett Research) with Sybr Green. The mtDNA was quantified by amplifying a 113 bp region of mitochondrial large ribosomal RNA gene (*lrRNA*, CR34094) with primer q13259F (5’-TCGTCCAACCATTCATTCC-3’) and q13371R (5’-ATAAAGTCTAACCTGCCCACTGA-3’). Nuclear genome DNA was quantified by amplifying a 268 bp region of the single-copy housekeeping gene *Rp49* using primer Rp49F (5’-AGATCGTGAAGAAGCGCACC-3’) and Rp49R (5’-CACCAGGAACTTCTTGAATC-3’). Each 10 μL of reaction mixture contains 1 μL of 5 ng DNA, 1 μL of each 2.5 μM forward and reverse primer, 2 μL of distilled water and 5 μL of Sybr Green. The quantitative PCR program included denaturing at 95°C for 5 min and amplification in 40 cycles of 95°C for 10 s followed by 60°C for 30 s. Amplification was followed by a melting curve from 72°C to 95°C, rising by steps of 0.5°C, to verify that a single product was amplified. The mtDNA copy number was expressed as mean mtDNA copy number relative to nuclear genome (*n* = 6 replicate/ line/ sex/ age/ diet).

#### Maximum reactive oxygen species (ROS) production

Mitochondrial Complex I is a major site of ROS production [57], and ROS levels may vary with diet [58]. H_2_O_2_ released from extracted mitochondria [59] was determined. H_2_O_2_ reacts with Ampliflu red (Sigma-Aldrich) in the presence of horseradish peroxidase to form the oxidative product resofurin, which has a maximum absorbance at 560 nm [60].

Maximum ROS production was determined in 96-well microplates, with each well containing 50 μL of 0.2 ng/ μL of mitochondria and 135 μL of respiration buffer. All measures were performed at 23°C. The reaction was started by adding 5 μL of Ampliflu red (2.20 mM) and 10 μL of horseradish peroxide (0.02U• μL^-1^). To determine the rate of maximum ROS production for Complex I, the substrate- inhibitor protocol was added sequentially as follows: pyruvate + malate + L-proline (each at 10 mM) and rotenone (0.5 μM). Results were recorded every 1 min for 15 min in each state using a SpectraMax Plus spectrophotometer and SoftMax Pro software (Molecular Devices). The amount of ROS production in each well was expressed as pmol of H_2_O_2_ production• min^-1^• mg^-1^ of protein ± SEM (*n*≥ 9 replicate/ line/ sex/ age/ diet).

#### Superoxide dismutase activity

SOD activity constitutes the first line of defense against ROS [61–63], and total activity was assayed. Briefly, SOD activity was estimated based on the reduction of water-soluble formazan dye (SOD Assay Kit, Sigma-Aldrich). The rate of reduction of water-soluble formazan dye is linearly related to xanthine oxidase activity and is inhibited by SOD. Therefore, SOD activity can be quantified by measuring the decrease in the color development of the dye at 440 nm. Experimental samples were prepared by grinding one thorax in 100 μL of ice cold buffer containing 100 mM KH_2_PO_4_, 1 mM EDTA and 0.1% triton X, pH 7.5). Three blanks were included in the assay (blank 1 without the sample, blank 2 without enzyme and blank 3 with only buffer). SOD activity was performed in 96-well microplates, with each well containing 20 μL of ground thorax in 200 μL of working buffer. All measures were performed at 23°C. The reaction was started by adding 20 μL of enzyme working solution into the well. The SOD activity rate was calculated as: (blank 1—blank 3)—(sample—blank 2)/ (blank 1—blank 3) • 100 and expressed as U• mg^-1^• fibre (*n*≥ 9 replicate/ line/ sex/ age/ diet).

### Physiological traits

#### Fecundity

It has previously been shown that females are more influenced by diet than males [64] and female fecundity is more influenced by age than is male fecundity [65, 66]. Here we conducted complimentary assays to distinguish mitotype and then diet and age affects. To identify mitotype effects, males and females of both mitotypes were mated in all possible permutations and fecundity of 10 males and 10 females assayed. All flies were placed in new vials containing fresh food every 24 h for 25 d. The number of eggs was counted daily, and fecundity was calculated as a total number of eggs from 1–11 d and 12–25 d (*n* = 10 replicate/ line/ sex/ age/ diet). This protocol is limited as we cannot distinguish the sex-specific effects of diet or age.

Next, we quantified male reproductive performance with a control cross. These assays included 10 males (11 d and 25 d of age) of both mitotypes fed either the 1:2 P:C or 1:16 P:C food and mated with 10 virgin 5 d old *Alst; w*^*1118*^ females fed the 1:6 P:C diet. Males were fed the 1:2 P:C or the 1:16 P:C diets as they span the study range. Females were fed the 1:6 P:C diet as it is intermediate. We included *Alst; w*^*1118*^ females as they do not harbor the ND2 mutation that we predicted was functionally important. After an 8 h mating period in vials containing 1% agar, females were transferred to a new vial containing fresh food. The number of eggs was counted daily for 6 d (*n* = 10 replicate/ line/ age/ diet). We did not assay female fecundity directly because virgin females produce eggs and older virgin females are less fecund [67].

#### Survival

Mitotype and diet are known to influence survival in *Drosophila* [25, 40]. Here, flies were sorted 24 h after eclosion and 10 males and 10 females placed together in 30-mL plastic vials containing 5 mL of one diet. Food vials were replaced every 3 d, and survival was recorded every 48 h until all flies were dead. The 50% survival was then calculated (*n* = 8 replicate/ line/ sex/ age/ diet).

#### Lipid content

In humans, it has been shown that mtDNA mutations and dietary sugar can cause an increase in lipid production [32, 68]. Here, lipid content was assayed following Hoffman and colleagues [69]. Briefly, flies were transferred into 1.5-mL Eppendorf tubes, frozen in liquid nitrogen for 1 min, and then stored at -80°C for 3 d. Flies were individually transferred into a single well of a 96-well microplate and dried in an oven at 60°C for 24 h. Flies were then weighed to the nearest 0.01 mg using a CP2P micro balance (Satorius) in groups of 8. Body lipids were then removed by placing flies into a polyethylene tea bag submerged in 10 mL of diethyl ether in a 30 mL glass tube for 24 h at room temperature. Parafilm was used to seal the glass tubes to avoid evaporation of diethyl ether. Flies were then dried in an oven at 60°C for 24 h and weighed again. The lipid content was calculated as the difference between dry weight and final weight after lipid extraction [69](*n* = 9 replicate/ line/ sex/ age/ diet).

#### Starvation resistance

We assayed starvation resistance as it is positively correlated with lipid levels [33] and tends to be greatest when fed a low P:C diet [70]. Briefly, 11 d and 25 d old females and males were separated on ice and then transferred into a vial containing 5 mL of 1% agar in groups of 10. Starvation resistance was measured by counting the surviving flies every 12 h until all flies had died. Starvation resistance was calculated as 50% survival (*n* = 8 replicate/ line/ sex/ age/ diet).

#### Statistical analysis

We analyzed each data set by ANOVA using JMP 12 Software (SAS Institute). The main effects were mitotype, diet, and age. The experimental blocks were treated as a random effect and significance determined using the method of Self and Liang [71]. Block was not significant in any assay and is excluded from subsequent reporting. For fecundity, data including males mated with females of the same mitotype and males mated with females of the alternate mitotype were combined. In this case, males and females were analyzed separately so that the effects of mitotype and their interactions could be determined. Throughout all remaining assays, there was a consistent influence of sex (S1 Table), and therefore we considered females and males separately (see Tables 1 and 2). The lipid content data were transformed using ARCSIN square root for ANOVA analysis.

**Table 1:** Mitochondrial function traits analyses of variance results.

Mitochondrial		Nuclear	Total	Mitotype	Diet	Age	M	M	D
function		Background		(M)	(D)	(A)	x	x	x
traits							D	A	A
			*n*	*df* = 1	*df* = 3	*df* = 1	*df* = 3	*df* = 3	*df* = 1
CI-OXPHOS									
	Male	*w*^*1118*^	96	43.12***	4.16*	343.66***	0.81	0.97	3.97*
	Female	*w*^*1118*^	96	43.51***	7.99***	317.93***	0.82	2.51	2.48
	Male	*OreR*	24	13.90**	N/A	4.776*	N/A	0.13	N/A
	Female	*OreR*	24	46.02***	N/A	12.70**	N/A	0.17	N/A
MtDNA Copy Number									
	Male	*w*^*1118*^	95	10.90*	20.37***	16.39**	2.08	2.75*	2.31*
	Female	*w*^*1118*^	93	1.50	3.03*	5.52*	1.44	1.04	0.60
Maximum ROS Production									
	Male	*w*^*1118*^	83	9.56**	5.55**	35.64***	0.87	1.27	10.99***
	Female	*w*^*1118*^	83	0.35	2.74*	56.50***	0.47	0.01	4.94**
SOD Activity									
Male		*w*^*1118*^	155	4.00*	8.03***	0.19	0.97	0.01	5.60*
Female		*w*^*1118*^	154	2.19	21.33***	0.36	1.88	2.35	8.77***

Data given as F-value

* P< 0.05

**P< 0.001

***P< 0.0001

**Table 2:** Physiological trait analyses of variance results.

Physiological		Nuclear	Total	Mitotype	Diet	Age	M	M	D
traits		Background		(M)	(D)	(A)	x	x	x
							D	A	A
			*n*	*df* = 3	*df* = 1	*df* = 1	*df* = 3	*df* = 3	*df* = 1
Fecundity									
	Male^a^	*w*^*1118*^	243	20.65***	1227.32***	905.93***	4.26*	18.68***	291.95***
	Female	*w*^*1118*^	243	0.35	1042.01***	769.14***	2.40	0.0008	247.87***
	Control^b^	*w*^*1118*^	96	4.70*	1.76	2.72	2.12	0.55	0.30
Survival									
	Male	*w*^*1118*^	62	6.52*	56.53***	N/A	3.23*	N/A	N/A
	Female	*w*^*1118*^	63	0.09	146.18***	N/A	0.10	N/A	N/A
Lipid Content									
	Male	*w*^*1118*^	144	4.39**	10.32**	0.20	0.15	`14.43**	0.04
	Female	*w*^*1118*^	144	3.02	124.94***	1.25	0.09	2.62	3.75*
Starvation Resistance									
	Male	*w*^*1118*^	128	7.23*	0.31	4.08*	0.47	14.56**	1.26
	Female	*w*^*1118*^	128	47.00***	173.25***	138.08***	1.08	7.47*	3.79*

Data given as F-value

* P< 0.05

**P< 0.001

***P< 0.0001.

^a^ Males mated with females of same mitotype and males mated with females of alternate mitotype

^b^ Males of each mitotype fed the 1:2 P:C and 1:16 P:C diets mated with 5 d old virgin *Alst; w*^*1118*^ females fed the 1:6 P:C diet.

## Results

### Mitotype differences

**Fig 1:**
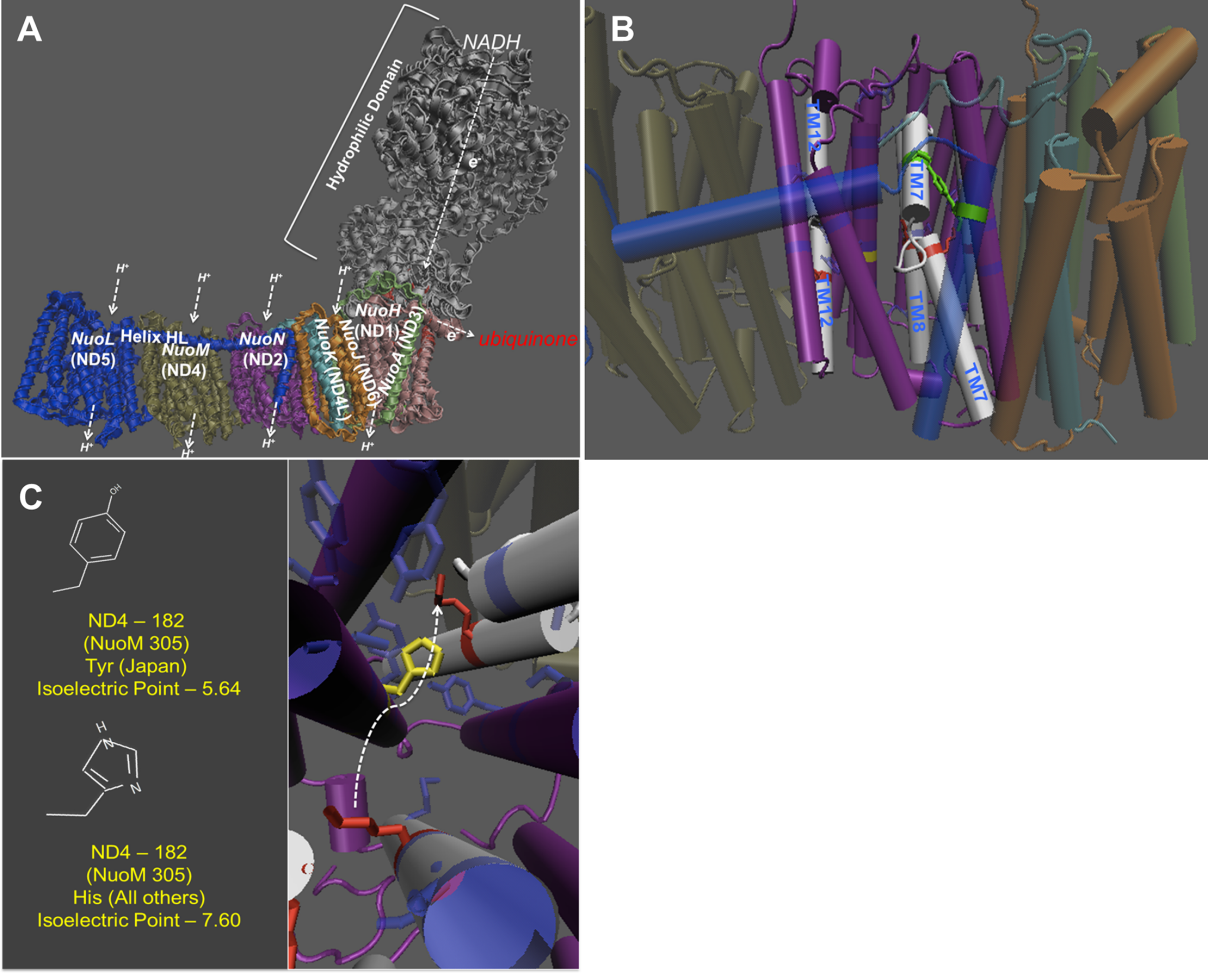
Complex I structure prediction of Japan mtDNA. (A) The ND2 region corresponds to the bacterial subunit NuoN. (B) The structure of the proton pump. The dark blue residues that are transparently shaded make up the proton channel. (C) The location of the variant and their isoelectric point. The yellow is the amino acid that is changed in the Japan line. The arrow represents the movement of the TM7 domain.

The putative His182Tyr ND2 substitution in Japan mtDNA is predicted to have slightly deleterious implications for proton movement through the ND2 pump. It corresponds to residue 305 in the NuoN subunit for *E*. *coli* (the large difference in residue numbering is the result of a deletion of the N-terminal portion of the ND2 subunit early in evolution) (Fig 1). This residue is highly conserved across diverse taxa and is a central component of the proton translocation channel [72]. Histidine provides an efficient means to transfer protons in general because it alternates between protonated (positively charged) and unprotonated (neutral) with subtle shifts in pH (isoelectric point = 7.60). Conversely, tyrosine is most likely to be deprotonated at physiological pH (isoelectric point = 5.64) and would therefore likely result in a proton sink.

No sequence differences in the A + T rich region were predicted to be functionally significant (S2 Table, Short read archive accession number: SRR6052098). PacBio long-read sequencing of the Alstonville and Japan mtDNA produced 882.56 Mb of raw data. Read lengths averaged 5,573 bases with longest read length of 24,822 bases and mean coverage of 40X for each mtDNA. There were no differences in length of the region (4626 nt), no difference in the number or length of Type I (5 repeats) or Type II (4 complete plus one partial) repeat elements and no difference in the length of the central T-stretch (21 nt). The A + T rich regions shared 99 percent sequence identity. Following Lewis and colleagues [47], there were 18 single nucleotide differences occurring in the Type I elements (2 in I-A, 5 in I-B1, 5 in I-C/ A, 5 in I-B2, and 1 in I-C) and 17 single nucleotide differences in the Type II elements (2 in II-A, 5 in II-B1, 6 in II-B2, 3 in II-C and 1 in the partial Type II element). No nucleotide differences occurred in the regions of potential secondary structure that were identified by Sugihara and colleagues [48].

### Mitochondrial function traits

#### CI-OXPHOS

**Fig 2:**
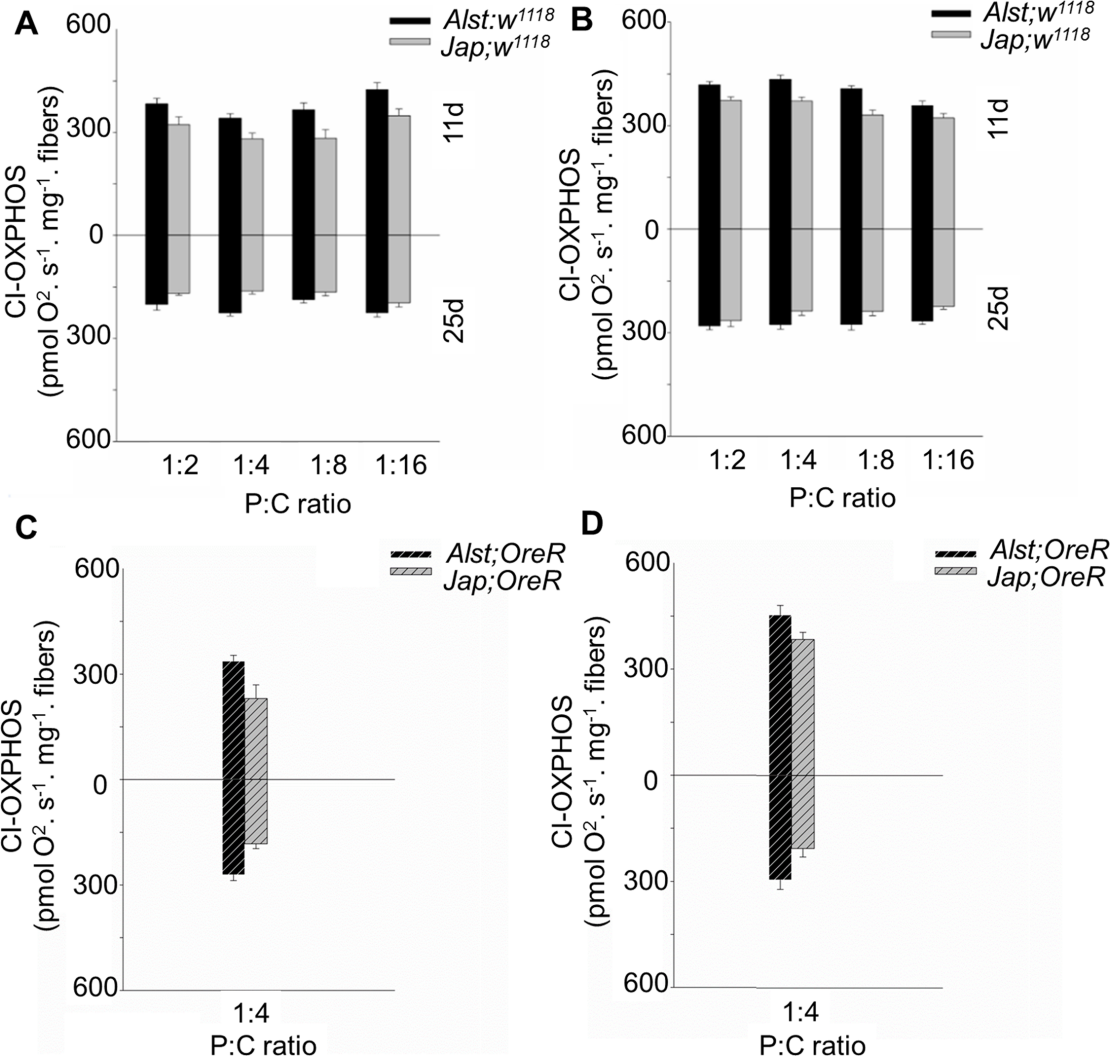
The mitochondrial CI-OXPHOS of *Drosophila melanogaster* harboring Alstonville (*Alst*) and Japan (*Jap*) mtDNA in the *w*^*1118*^ genetic background and the Oregon R (*OreR*) background. Flies were aged 11 (upper chart) and 25 d (lower chart). The Protein: Carbohydrate (P:C) diets were 1:2, 1:4, 1:8 and 1:16. (A) Males with the *w*^*1118*^ genetic background. (B) Females with the *w*^*1118*^ genetic background. (C) Males with the *OreR* genetic background. (D) Females with the *OreR* genetic background. Bar represents oxygen flux per mass, and error bars show the standard error of the mean.

CI-OXPHOS respiration rate of permeabilized fibers was used to determine the functionality of mitochondrial Complex I. Consistent with the known putative His182Tyr ND2 variant, flies with Japan mtDNA in the *w*^*1118*^ background had lower CI-OXPHOS respiration rates (Fig 2A and 2B). Further, young males and females had similar CI-OXPHOS respiration rates (Fig 2A and 2B upper half of graph), but males had a lower CI-OXPHOS than females as they age (Fig 2A and 2B lower half of graph). For males, ANOVA reported significant main effects of mitotype, diet, age and a diet by age interaction (Table 1). For females, ANOVA detected significant main effects of mitotype, diet, and age (Table 1).

To determine whether the lower CI-OXPHOS respiration rates in Japan males was caused by the putative His182Tyr ND2 mtDNA mutation or a disrupted mito-nuclear interaction, we assayed flies harboring the *OreR* nuclear background. The *Alst; OreR* and *Jap; OreR* flies showed the same trend as *Alst; w*^*1118*^ and *Jap; w*^*1118*^ flies (Fig 2C and 2D, Table 1). Statistical analyses using ANOVA, with main effects of mitotype (M) and nuclear background (N) in the 1:4 P:C treatment reported a significant effect of mitotype in males (M: F_1,23_ = 22.68, P< 0.001) and females (M: F_1,23_ = 26.41, P< 0.001). There were no significant effects of nuclear background or mito-nuclear interaction in either males (N: F_1,23_ = 0.32, P = 0.57; M x N: F_1,23_ = 2.55, P = 0.12) or females (N: F_1,23_ = 0.54, P = 0.47; M x N: F_1,23_ = 0.66, P = 0.42). As the primary difference occurred between mitotypes, subsequent studies focused on mitotypes in the *w*^*1118*^ nuclear genetic background.

#### Mitochondrial DNA copy number

**Fig 3:**
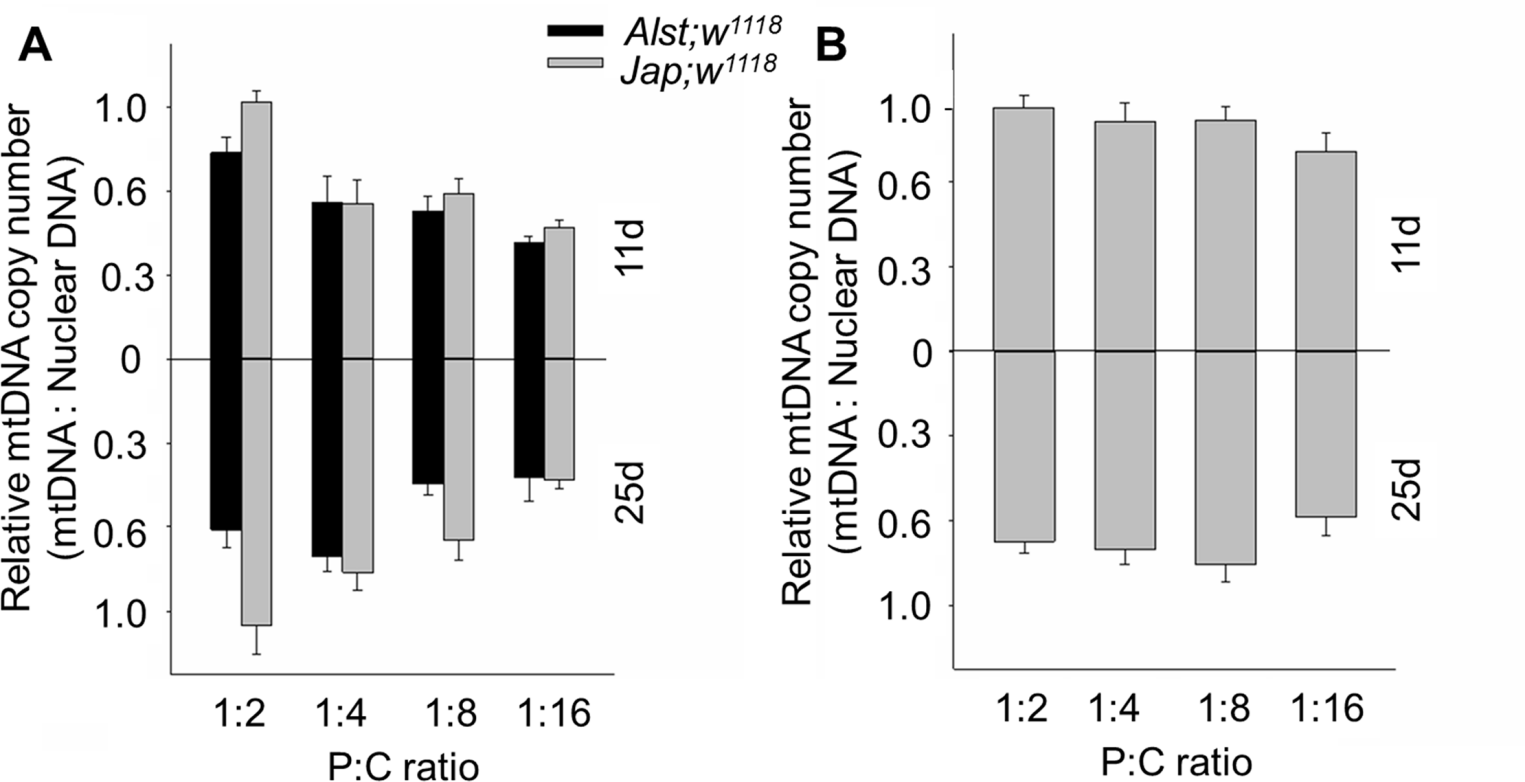
The mtDNA copy number of *Drosophila melanogaster* harboring Alstonville (*Alst*) and Japan (*Jap*) mtDNA in the *w*^*1118*^ genetic background. Flies were aged 11 (upper chart) and 25 d (lower chart). The protein: carbohydrate (P:C) diets were 1:2, 1:4, 1:8 and 1:16. (A) Males. (B) Females. MtDNA copy number of females harboring the two mtDNA types did not differ, so they were pooled. Data for each mitotype is presented in S1 Fig. Bar represents mtDNA copy number, and error bars show the standard error of the mean.

Mildly deleterious mtDNA mutations can influence copy number as they may be compensated through mtDNA over-replication [56, 73]. For males, *Jap; w*^*1118*^ had a higher mtDNA copy number than *Alst; w*^*1118*^ (Fig 3A) suggesting that the former flies compensate for the slightly deleterious His182Tyr ND2. ANOVA detected significant main effects of mitotype, diet, and age as well as mitotype by age and diet by age interactions (Table 1). For females, the mtDNA copy number of flies was lowest at 25 d of age (Fig 3B). ANOVA showed significant main effects of diet and age (Table 1). ANOVA shows no significant effect of mitotype.

#### Maximum reactive oxygen species production

**Fig 4:**
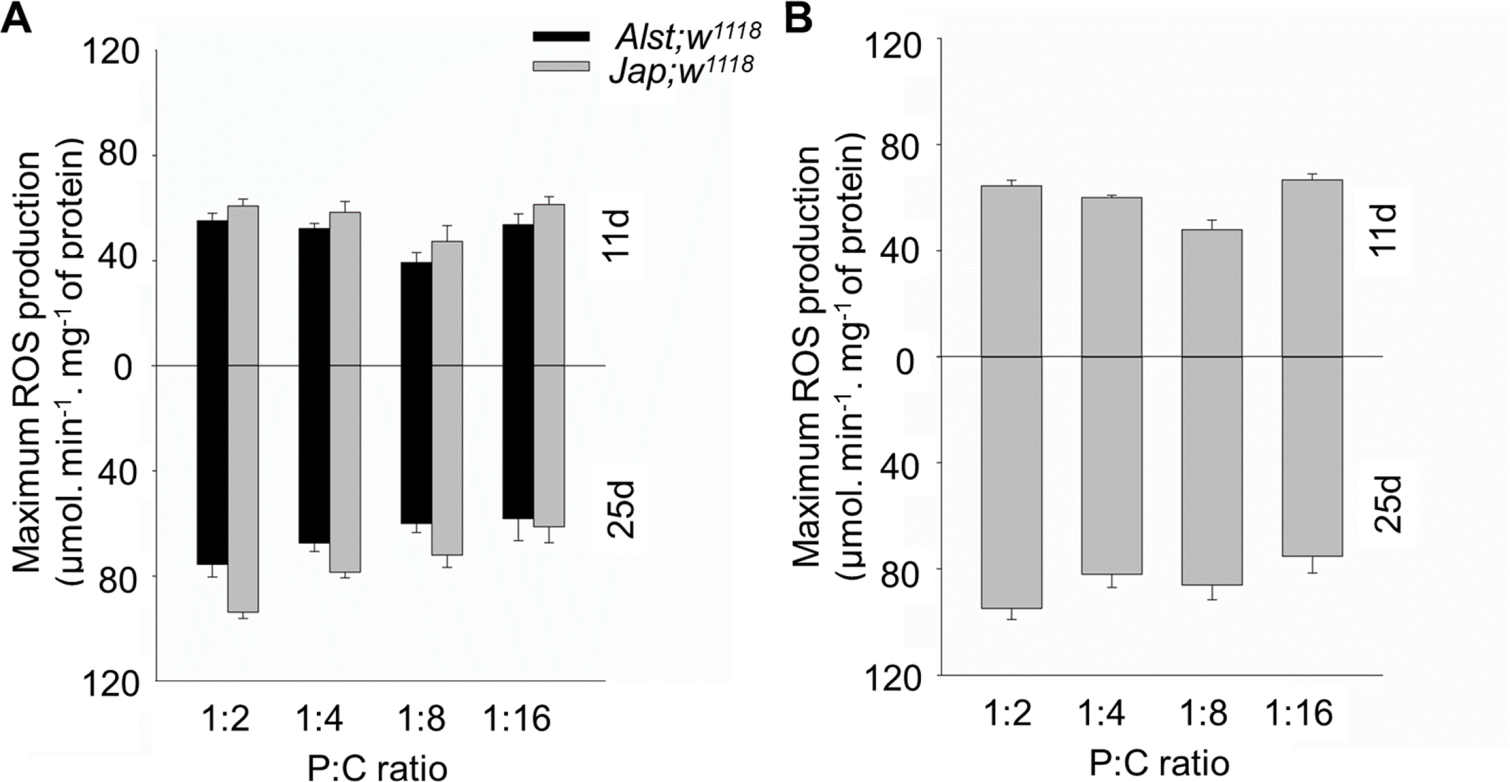
The maximum ROS production of *Drosophila melanogaster* harboring Alstonville (*Alst*) and Japan (*Jap*) mtDNA in the *w*^*1118*^ genetic background. Flies were aged 11 (upper chart) and 25 d (lower chart). The protein: carbohydrate (P:C) diets were 1:2, 1:4, 1:8 and 1:16. (A) Males. (B) Females. Maximum ROS production of female flies harboring the two mtDNA types did not differ, so they were pooled. Data for each mitotype is presented in S2 Fig. Bar represents basal ROS production, and error bars show the standard error of the mean.

ROS levels are also known to be influenced by diet and age [14, 56]. We predicted levels would be higher in Japan flies harboring the putative His182Tyr ND2 substitution as Complex I is a major source of ROS [74]. For both sexes, maximum ROS production in younger flies was lowest when fed 1:8 P:C ratio but highest when fed 1:2 and 1:16 P:C ratio food. Combined this is suggestive of an underlying metabolic shift in ROS production (Fig 4A and 4B). For males, maximum ROS production was higher in *Jap; w*^*1118*^ than in *Alst; w*^*1118*^. In older males, ROS production in both lines decreased with decreasing P:C ratio (Fig 4A, lower panel). ANOVA described significant main effects of mitotype, diet, and age and a diet by age interaction (Table 1). In older females, maximum ROS production was highest when fed the 1:2 P:C ratio diet and lowest the 1:16 P:C ratio food (Fig 4B). ANOVA reported significant effects of diet, age and a diet by age interaction but no significant effect of mitotype (Table 1).

#### Superoxide dismutase activity

**Fig 5:**
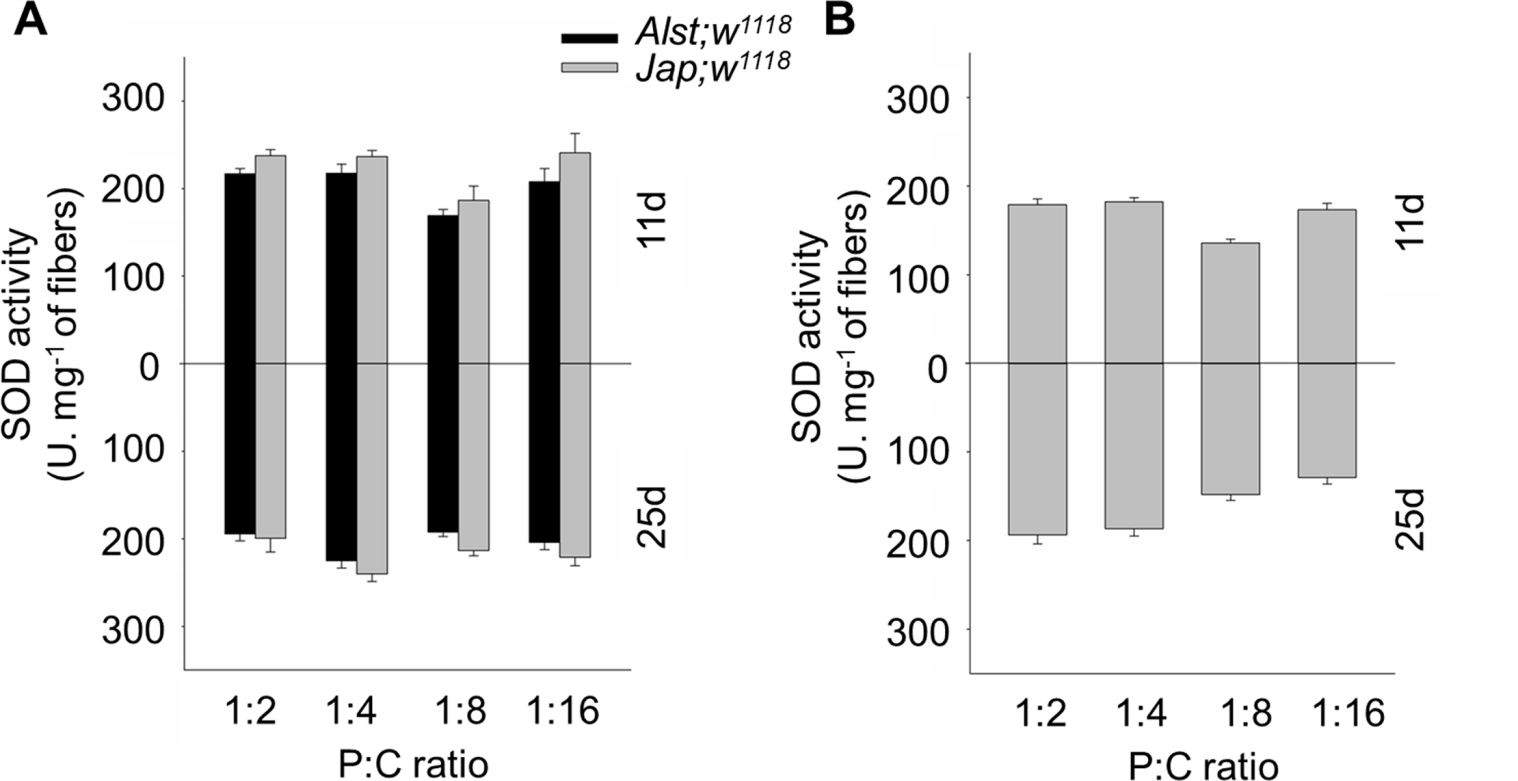
The SOD activity of *Drosophila melanogaster* harboring Alstonville (*Alst*) and Japan (*Jap*) mtDNA in *w*^*1118*^ genetic background. Flies were aged 11 (upper chart) and 25 d (lower chart). The protein: carbohydrate (P:C) diets were 1:2, 1:4, 1:8 and 1:16. (A) Males. (B) Females. The SOD activity of females harboring the two mtDNA types did not differ, so they were pooled. Data for each mitotype is presented in S3 Fig. Bar represents SOD activity, and error bars the standard error of the mean.

SOD is an important antioxidant defense and is expected to be higher in more oxidatively stressed flies. Overall, males had a higher SOD activity than females (Fig 5A and 5B). For both sexes, younger flies fed the 1:8 P:C diet have the lowest SOD activity further supporting the hypothesis that diet mediates an underlying metabolic shift. As expected, SOD activity was higher in *Jap; w*^*1118*^ males (Fig 5A). ANOVA showed significant mitotype, diet, and diet by age effects (Table 1). In older females, SOD activity decreased with decreasing P:C ratio (Fig 5B). ANOVA reported significant effects of diet and a diet by age interaction (Table 1).

### Physiological traits

#### Fecundity

**Fig 6:**
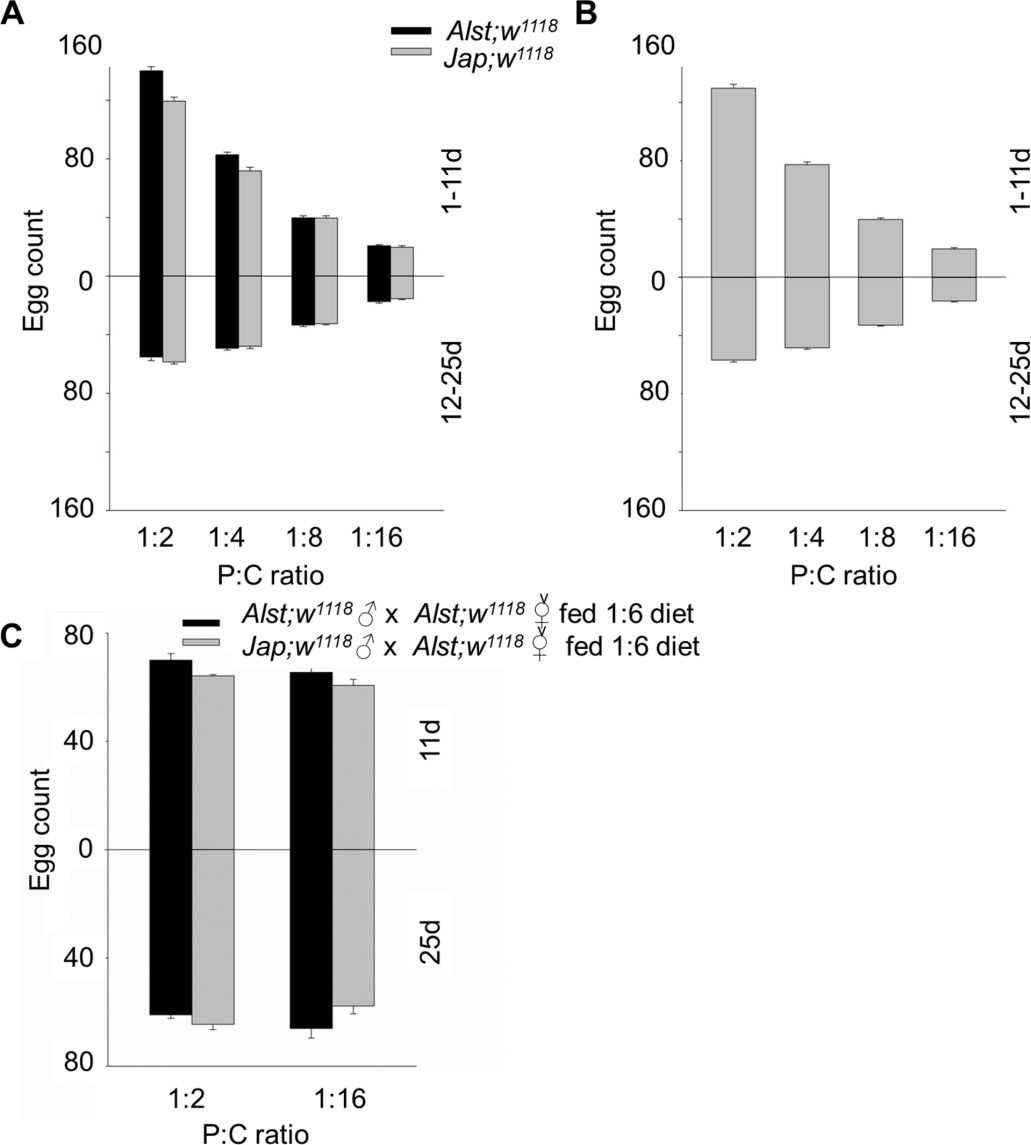
The fecundity of *Drosophila melanogaster* harboring Alstonville (*Alst*) and Japan (*Jap*) mtDNA in *w*^*1118*^ genetic background. The flies were aged from 1–11 d (upper chart) and 12–25 d (lower chart). The protein: carbohydrate (P:C) diets were 1:2, 1:4, 1:8 and 1:16. (A) Males. (B) Females. (C) The fecundity of males (11 d or 25 d) mated with 5 d old virgin females fed on the intermediate 1:6 P:C diet. Data for each mitotype is presented in S4 Fig. Bar represents total egg count, and error bars show standard error of the mean.

Parental investment per gamete is considered to be negligible for males but a primary bioenergetic cost for females [64]. As a consequence, we predicted females would be more sensitive to dietary manipulation [64–66]. From our data, the most noticeable trend is that the number of eggs produced declined with decreasing P:C ratio, but the decline was more distinct in young flies (Fig 6A and 6B). For males, younger *Alst; w*^*1118*^ flies have a higher fecundity than *Jap; w*^*1118*^ flies but the fecundity for older flies harboring each mitotype did not differ (Fig 6A). ANOVA showed a significant effect of mitotype, diet, age, mitotype by diet, mitotype by age and a diet by age interaction (Table 2). For females, there was no mitotype effect. ANOVA reported a significant effect of diet, age, and diet by age interaction (Table 2). It is most likely that these are female effects [64–66], however, we wished to test this hypothesis.

To determine the reproductive performance of males we fed each mitotype the 1:2 P:C or the 1:16 P:C diet and crossed them with 5 d old virgin *Alst; w*^*1118*^ females fed a constant 1:6 P:C diet. Consistent with our previous results, young Japan males mated to control females produced fewer eggs than did Alstonville males (Fig 6A and 6C). ANOVA reported significant effects of mitotype but no significant effects of diet or age (Table 2). As there were no significant diet or age effects in male reproductive performance, we suggest these results support the hypothesis that female fecundity is influenced by diet and age [64–66].

#### Survival

**Fig 7:**
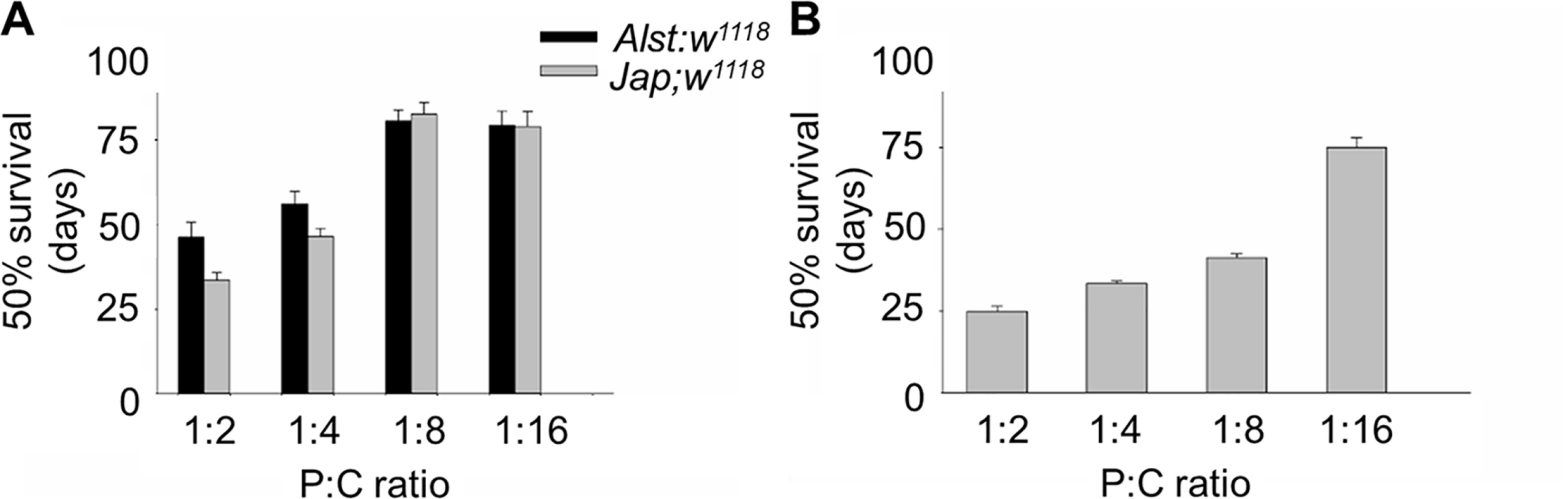
The survival of *Drosophila melanogaster* harboring Alstonville (*Alst*) and Japan (*Jap*) mtDNA in *w*^*1118*^ genetic background. The protein: carbohydrate (P:C) diets were 1:2, 1:4, 1:8 and 1:16. (A) Males. (B) Females. Survival of females harboring the two mtDNA types did not differ, so they were pooled for graphical presentation. Data for each mitotype is presented in S5 Fig. Bar represents 50% survival, and error bars show standard error of the mean.

As previously shown for mated females [25, 75] survival increased with decreasing P:C ratio when females and males are housed together (Fig 7A and 7B). This trend is less evident in males. For males, *Jap; w*^*1118*^ flies tend to have lower survival than *Alst; w*^*1118*^ when fed with 1:2 P:C and 1:4 P:C diets (Fig 7A). ANOVA reported significant effects of mitotype, diet, and mitotype by diet (Table 2). For females, mean survival tended to be lower than males, with a plateau of ~ 80 d occurring on the 1:8 P:C and 1:16 P:C diets (Fig 7B). ANOVA showed a significant effect of diet but not mitotype (Table 2).

#### Lipid content

**Fig 8:**
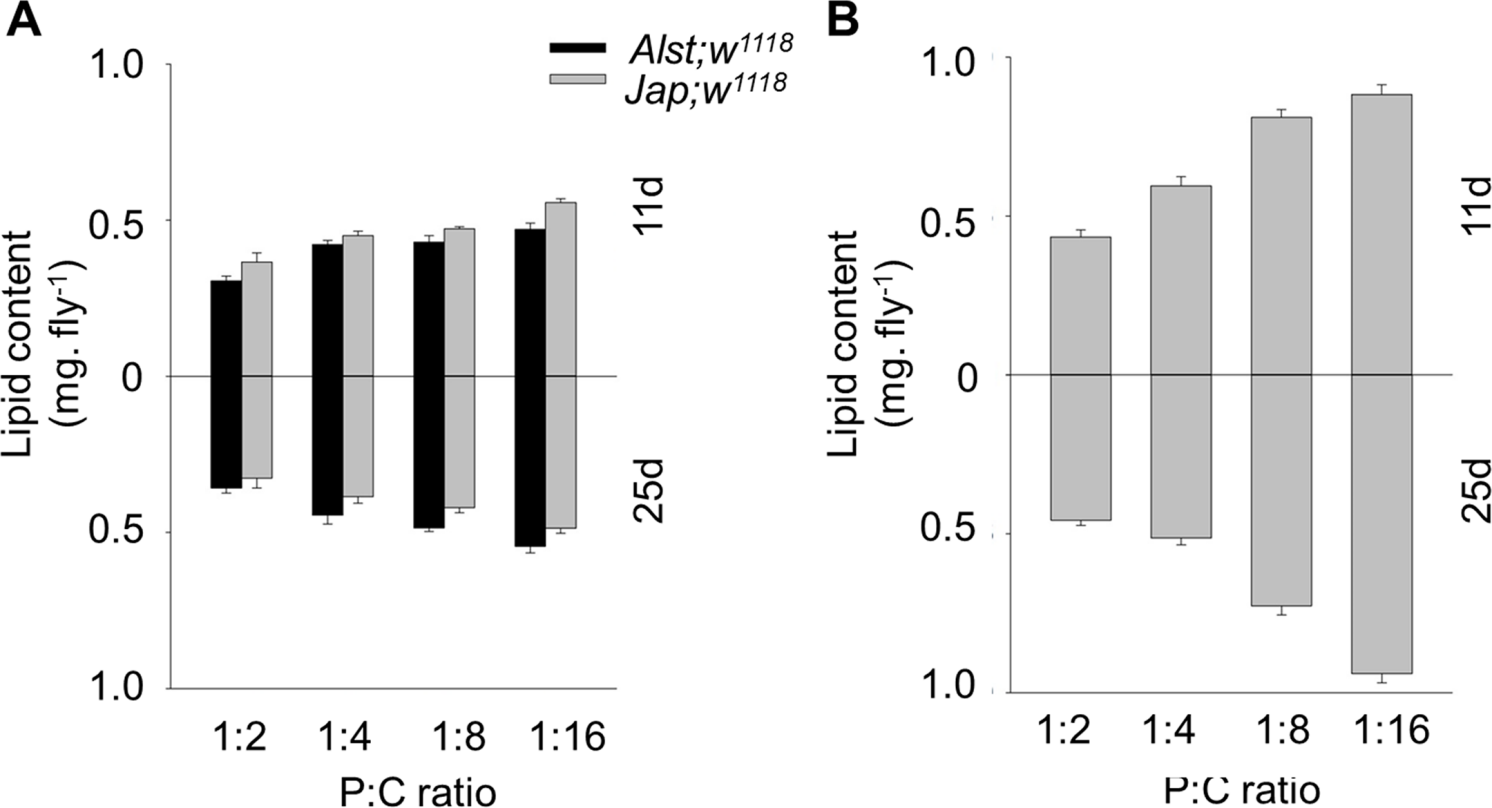
The lipid content of *Drosophila melanogaster* harboring Alstonville (*Alst*) and Japan (*Jap*) mtDNA in *w*^*1118*^ genetic background. Flies were aged 11 d (upper chart) and 25 d (lower chart). The protein: carbohydrate (P:C) diets were 1:2, 1:4, 1:8 and 1:16. (A) Male. (B) Female. Lipid content of females harboring the two mtDNA types did not differ, so they were pooled. Data for each mitotype is presented in S6 Fig. Bar represents lipid content, and error bars show standard error of the mean.

Lipid content was assayed because it is influenced by sex, mtDNA mutations and dietary change [68, 70, 76]. Overall, lipid content was less influenced by diet in males than in females (Fig 8A and 8B). For males, lipid content of young *Jap; w*^*1118*^ flies was higher than *Alst; w*^*1118*^ flies but the reverse trended to be true for older males (Fig 8A). ANOVA reported significant effects of mitotype, diet, and mitotype by age (Table 2). Females’ lipid content increased with decreasing P:C ratio at both ages (Fig 8B). ANOVA showed significant effects of diet and a diet by age interaction (Table 2). There was no significant effect of mitotype (Table 2).

#### Starvation resistance

**Fig 9:**
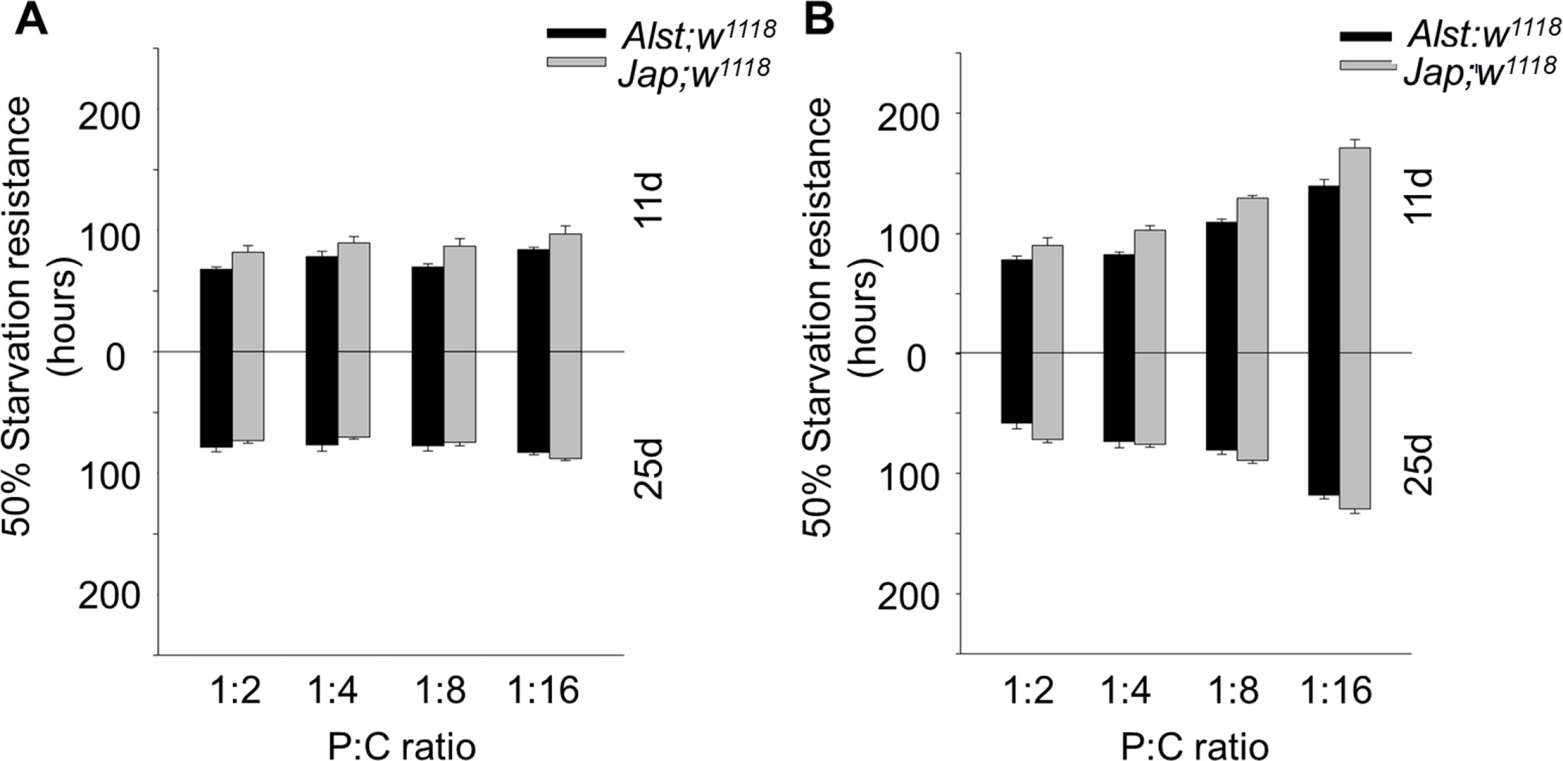
The starvation resistance of *Drosophila melanogaster* harboring Alstonville (*Alst*) and Japan (*Jap*) mtDNA in *w*^*1118*^ genetic background. Flies were aged 11 (upper chart) and 25 d (lower chart). The protein: carbohydrate (P:C) diets were 1:2, 1:4, 1:8 and 1:16. (A) Males. (B) Females. Bar represents 50% survival, and error bars show standard error of the mean.

Starvation resistance is positively correlated with lipid levels and confers resistance to stress in nature [33, 70]. As predicted from the lipid data, males were less influenced by diet than females (Fig 9A and 9B). For males, the starvation resistance of young *Jap; w*^*1118*^ males was higher than *Alst; w*^*1118*^ males while the resistance of older males from both lines was similar (Fig 9A). ANOVA showed significant effects of mitotype, age, and mitotype by age (Table 2). For females, mean starvation resistance increased by ~ 40% with decreasing P:C ratio. Furthermore, *Jap; w*^*1118*^ females tended to have greater resistance than *Alst; w*^*1118*^ females (Fig 9B). ANOVA detected significant main effects of mitotype, diet, and age, as well as mitotype by age and diet by age interactions (Table 2).

## Discussion

Sex-specific differences in energy metabolism are likely the consequence of the interplay between maternally inherited mitochondria and sex chromosomes as well as differences in endocrine activity, including juvenile hormone and ecdysone. In this study, we identify the sex-specific role of mtDNA and diet in two *D*. *melanogaster* fly lines. MtDNA mutations tend to have larger effects on males than females. This result is in accord with the Mother's Curse hypothesis suggesting an accumulation of male-harming mutation in mtDNA [1, 5]. In contrast, diet affects females more than males. We posit this result from greater metabolic flexibility in females that is tuned to the evolutionary and metabolic functions required by females for reproduction.

For males, the influence of mitotype predominates with our data showing that flies harboring Japan mtDNA with the putative His182Tyr change in ND2 have reduced mitochondrial functions. The Complex I structural analysis predicts the Japan mitotype will have reduced proton pumping and bioenergetic efficiency (Fig 1). Those males harboring Japan mtDNA have lower respiration of mitochondrial Complex I (Fig 2A and 2C), elevated mtDNA copy number (Fig 3A), higher maximum ROS (Fig 4A) and higher SOD activity (Fig 5A). Lower CI-OXPHOS and increased mtDNA copy number and ROS production reflect reduced complex function [77] while the higher SOD activity reflects increased oxidative stress [78]. The hypothesis that the Japan mitotype has reduced mitochondrial functions is supported by studies in humans where increased ROS production induced by mitochondrial dysfunction stimulates an increase in mtDNA copy number [79]. The higher mtDNA copy number may result in more respiratory complexes [80], increased mitochondrial mass [79] and, this may reflect a compensentory mechanism for mitochondrial dysfunction [81].

Males carrying the Japan mitotype also have reduced physiological functions. In accord with our previous finding [35], males harboring Japan mtDNA have reduced longevity when they are fed the higher protein diets (1:2 P:C and 1:4 P:C ratio) (Fig 7A). Likely, the sex-specific mitotype effects observed in our study are mediated by sexual dimorphism in mitochondria-mediated gene expression [82]. These results are consistent with the findings of Camus and colleagues [83] who showed the patterns of longevity and aging of virgin flies are more sensitive to mtDNA haplotype variation in males than in females. It also supports work from David Rand’s group showing that mtDNA might have a different fitness effect in males versus females [84]. Mitochondrial dysfunction has previously been associated with a large variety of diseases, such as insulin resistance, type 2 diabetes, reduced fertility, obesity, dyslipidemia, mitochondrial encephalomyopathy, lactic acidosis and stroke-like episodes, autism spectrum disorder and Leber hereditary optic neuropathy (LHON) [85–91]. Among these diseases, LHON is perhaps the best known example of a maternally inherited mitochondrial disease that affects males more severely than females [92]. Males also show a higher prevalence of diabetes, obesity and dyslipidemia than females with the links between these diseases and mitochondrial metabolism vigorously debated [93, 94].

In this study, we observe that females were more metabolically flexible and responsive toward dietary changes than males (Figs 6, 7, 8 and 9). A higher metabolic flexibility in females harboring the Japan mitotype may also enable increased ability to compensate for the putative His182Tyr ND2 mutation. Females with Japan mtDNA had lower CI-OXPHOS (Fig 2B) than those harboring Alstonville mtDNA, but the mitotypes had similar mtDNA copy numbers, ROS levels, and SOD activity (Figs 3B, 4B and 5B). Combined these data suggest females with Japan mtDNA functionally compensate for the mutation and are less stressed than males of the same mitotype. Intriguingly, Camus and colleagues [95] found large differences in mtDNA copy number between females harboring the Alstonville and Japan mitotypes, which is not evident in this study. This could be due to differences in experimental design as they employed whole flies fed on an unknown diet whereas ours was conducted on thoraces. This ability to compensate for deleterious mutations opens the door for dietary manipulations to be included as an integrated strategy to treat humans with mitochondrial diseases [96].

The greater metabolic flexibility of females likely results from their reproductive demands. Females fed the high P:C ratio diet have maximal egg production while those fed the low P:C diet have the maximal lifespan (Figs 6B and 7B). Remarkably, this change in reproductive investment links with sex-specific effects in lipid content and starvation resistance (Figs 8 and 9). In *Drosophila* and Queensland fruit flies, lifespan has previously been shown to increase as P:C ratios decreased while egg production was maximized on high P:C ratios [17, 25, 97]. Brooks and Garratt [98] reviewed the origins of sex-dependent aging and provide extensive evidence that reproduction, and the physiological pathways enabling reproduction, provide novel insight into the evolution of sex-specific differences in survival. As with flies, sex-specific effects in lipid content have also been linked with diet in humans and crickets [99, 100]. In humans, sex-specific specialization in energy metabolism is associated with distinct body-fat distribution and energy substrate-utilization patterns. Females store more lipids and have higher insulin sensitivity than males, while males tend to oxidize more lipids than females. Likely, these patterns are influenced by gestation and lactation in females and nutritional status and exercise intensity in both sexes [99]. One way to test whether greater metabolic flexibility in females results from reproductive demands is to contrast carbohydrate metabolism and mitochondrial bioenergetics in virgin and non-virgin flies fed diets that differ in their P:C ratio.

The sex-specific differences in energy metabolism have motivated evolutionary and metabolomic studies. In this study, we have shown the effect of mitotype is stronger in males than females inferring accumulation of male-harming mutations in mtDNA. In contrast, we show that diet has a stronger influence on females than males, and this is consistent with higher metabolic flexibility in the former. This study highlights the importance of including both males and females in studies associated with energy metabolism as there are unequal influences on each sex. Further research is needed to determine the molecular mechanims by which females switch from a reproductive focused mode to a survival mode.

## Supporting information

S1 Table Analyses of variance results showing F ratios. (DOCX)

S2 Table Nucleotide differences in the tandem repeats of the mitochondrial A + T rich region. Differences between Alstonville and Japan are compared to the nucleotide of *D*. *melanogaster* line Oregon R at the same position (GenBank: U11584.1). Positions are numbered from the start of the A + T rich region. Identity to Oregon R is indicated by (•) and gaps are denoted by (-).
(DOCX)

S1 Fig The mtDNA copy number of *Drosophila melanogaster* harboring Alstonville (*Alst*) and Japan (*Jap*) mtDNA in the *w*^*1118*^ genetic background. Flies were aged 11 (upper chart) and 25 d (lower chart). The protein: carbohydrate (P:C) diets were 1:2, 1:4, 1:8 and 1:16. (A) Males. (B) Females. Bar represents mtDNA copy number, and error bars show the standard error of the mean.
(TIF)

S2 Fig The maximum ROS production of *Drosophila melanogaster* harboring Alstonville (*Alst*) and Japan (*Jap*) mtDNA in the *w*^*1118*^ genetic background. Flies were aged 11 (upper chart) and 25 d (lower chart). The protein: carbohydrate (P:C) diets were 1:2, 1:4, 1:8 and 1:16. (A) Males. (B) Females. Bar represents basal ROS production, and error bars show the standard error of the mean.
(TIF)

S3 Fig The SOD activity of *Drosophila melanogaster* harboring Alstonville (*Alst*) and Japan (*Jap*) mtDNA in *w*^*1118*^ genetic background. Flies were aged 11 (upper chart) and 25 d (lower chart). The protein: carbohydrate (P:C) diets were 1:2, 1:4, 1:8 and 1:16. (A) Males. (B) Females. Bar represents SOD activity, and error bars the standard error of the mean.
(TIF)

S4 Fig The fecundity of *Drosophila melanogaster* harboring Alstonville (*Alst*) and Japan (*Jap*) mtDNA in *w*^*1118*^ genetic background. The flies were aged from 1–11 d (upper chart) and 12–25 d (lower chart). The protein: carbohydrate (P:C) diets were 1:2, 1:4, 1:8 and 1:16. (A) Males. (B) Females. (C) The fecundity of males (11 d or 25 d) mated with 5 d old virgin females fed on the intermediate 1:6 P:C diet. Bar represents total egg count, and error bars show standard error of the mean.
(TIF)

S5 Fig The survival of *Drosophila melanogaster* harboring Alstonville (*Alst*) and Japan (*Jap*) mtDNA in *w*^*1118*^ genetic background. The protein: carbohydrate (P:C) diets were 1:2, 1:4, 1:8 and 1:16. (A) Males. (B) Females. Bar represents 50% survival, and error bars show standard error of the mean.
(TIF)

S6 Fig The lipid content of *Drosophila melanogaster* harboring Alstonville (*Alst*) and Japan (*Jap*) mtDNA in *w*^*1118*^ genetic background. Flies were aged 11 d (upper chart) and 25 d (lower chart). The protein: carbohydrate (P:C) diets were 1:2, 1:4, 1:8 and 1:16. (A) Male. (B) Female. Bar represents lipid content, and error bars show standard error of the mean.
(TIF)
