# Supplementary material for: Sex-specific influences of mtDNA mitotype and diet on mitochondrial functions and physiological traits in Drosophila melanogaster
Source: PLoS One. 2017 Nov 22;12(11):e0187554. doi: 10.1371/journal.pone.0187554 (PMC5699850; doi:10.1371/journal.pone.0187554)
Supplement: S1 Table — (DOCX) [file pone.0187554.s001.docx]

|  | | Total | Sex | Mitotype | Diet | Age | S | S | S | M | M | D |
| --- | --- | --- | --- | --- | --- | --- | --- | --- | --- | --- | --- | --- |
|  | |  |  |  |  |  | x | x | x | x | x | x |
|  | |  |  |  |  |  | M | D | A | D | A | A |
|  | | *n* | *df*= 1 | *df*= 1 | *df*= 3 | *df*= 1 | *df*= 3 | *df*= 3 | *df*= 3 | *df*= 3 | *df*= 1 | *df*= 3 |
| **Mitochondrial Function Traits** | |  |  |  |  |  |  |  |  |  |  |  |
|  | CI-OXPHOS | 192 | 94.54^***^ | 80.29^***^ | 2.47 | 616.41^***^ | 1.28 | 8.08^***^ | 13.48^**^ | 0.60 | 2.88 | 1.87 |
|  | MtDNA Copy Number | 188 | 13.56^*^ | 9.39^**^ | 15.98^***^ | 20.07^***^ | 1.04 | 5.45^*^ | 2.57 | 3.00^*^ | 3.00^*^ | 1.42 |
|  | Basal ROS | 297 | 57.70^***^ | 3.12 | 98.72^***^ | 263.62^***^ | 0.12 | 0.67 | 0.63 | 0.53 | 0.50 | 44.89^***^ |
|  | SOD Activity | 308 | 140.17^***^ | 0.57 | 21.14^***^ | 0.54 | 7.69^*^ | 4.50^*^ | 0.01 | 1.77 | 2.29 | 6.80^**^ |
| **Physiological Traits** | |  |  |  |  |  |  |  |  |  |  |  |
|  | Lipid Content | 289 | 94.22^***^ | 6.83^**^ | 137.24^***^ | 0.52 | 0.30 | 51.34^***^ | 1.34 | 0.09 | 37.83^***^ | 2.83^*^ |
|  | Starvation Resistance | 256 | 173.02^***^ | 45.41^***^ | 122.20^***^ | 95.08^***^ | 9.00^*^ | 59.22^***^ | 49.19^***^ | 1.40 | 21.10^***^ | 2.06 |
|  | Longevity | 125 | 154.32^***^ | 4.81^*^ | 142.17^***^ | N/A | 3.71 | 2.86 | N/A | 2.63 | N/A | N/A |

**S1 Table**: Analyses of variance results showing F ratios.

Data given as F-value * P< 0.05, **P< 0.001, ***P< 0.0001.
