# Supplementary material for: Sex-specific influences of mtDNA mitotype and diet on mitochondrial functions and physiological traits in Drosophila melanogaster
Source: PLoS One. 2017 Nov 22;12(11):e0187554. doi: 10.1371/journal.pone.0187554 (PMC5699850; doi:10.1371/journal.pone.0187554)
Supplement: S2 Table — Differences between Alstonville and Japan are compared to the nucleotide of D. melanogaster line Oregon R at the same position (GenBank: U11584.1). Positions are numbered from the start of the A + T rich region. Identity to Oregon R is indicated by (•) and gaps are denoted by (-). (DOCX) [file pone.0187554.s002.docx]

**S2 Table.** Nucleotide differences in the tandem repeats of the mitochondrial A + T rich region. Differences between Alstonville and Japan are compared to the nucleotide of *D. melanogaster* line Oregon R at the same position (GenBank: U11584.1). Positions are numbered from the start of the A + T rich region. Identity to Oregon R is indicated by (•) and gaps are denoted by (-).

Position 111111111111122222233333333333444

77111334566678900167822222677777012

77119243368887322407144447900233970

01571207410237768433001897908902320

Oregon R CAAAAATAAT-TTAA-TTA-TTT--AAA-TATT-T

Alstonville TG••••••TATAA••A•A•AAAATTTT•T••••••

Japan A•TTTTAG••A••TGTA•TT•••AA••TAATACT-
