# Supplementary figures and images for: Sex-specific influences of mtDNA mitotype and diet on mitochondrial functions and physiological traits in Drosophila melanogaster
Source: PLoS One. 2017 Nov 22;12(11):e0187554. doi: 10.1371/journal.pone.0187554 (PMC5699850; doi:10.1371/journal.pone.0187554)

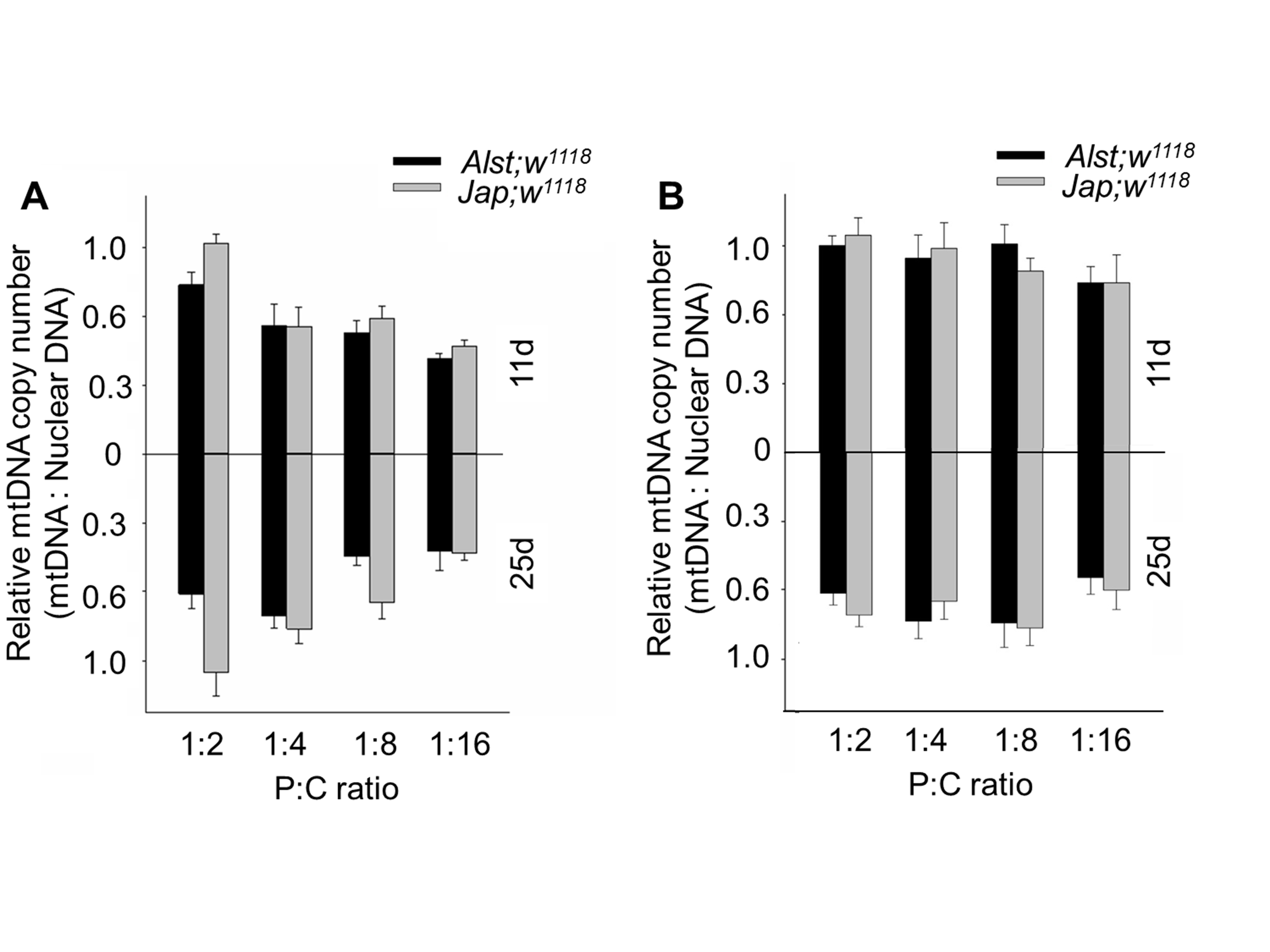

Supplement: S1 Fig — Flies were aged 11 (upper chart) and 25 d (lower chart). The protein: carbohydrate (P:C) diets were 1:2, 1:4, 1:8 and 1:16. (A) Males. (B) Females. Bar represents mtDNA copy number, and error bars show the standard error of the mean. (TIF) [file pone.0187554.s003.tif]

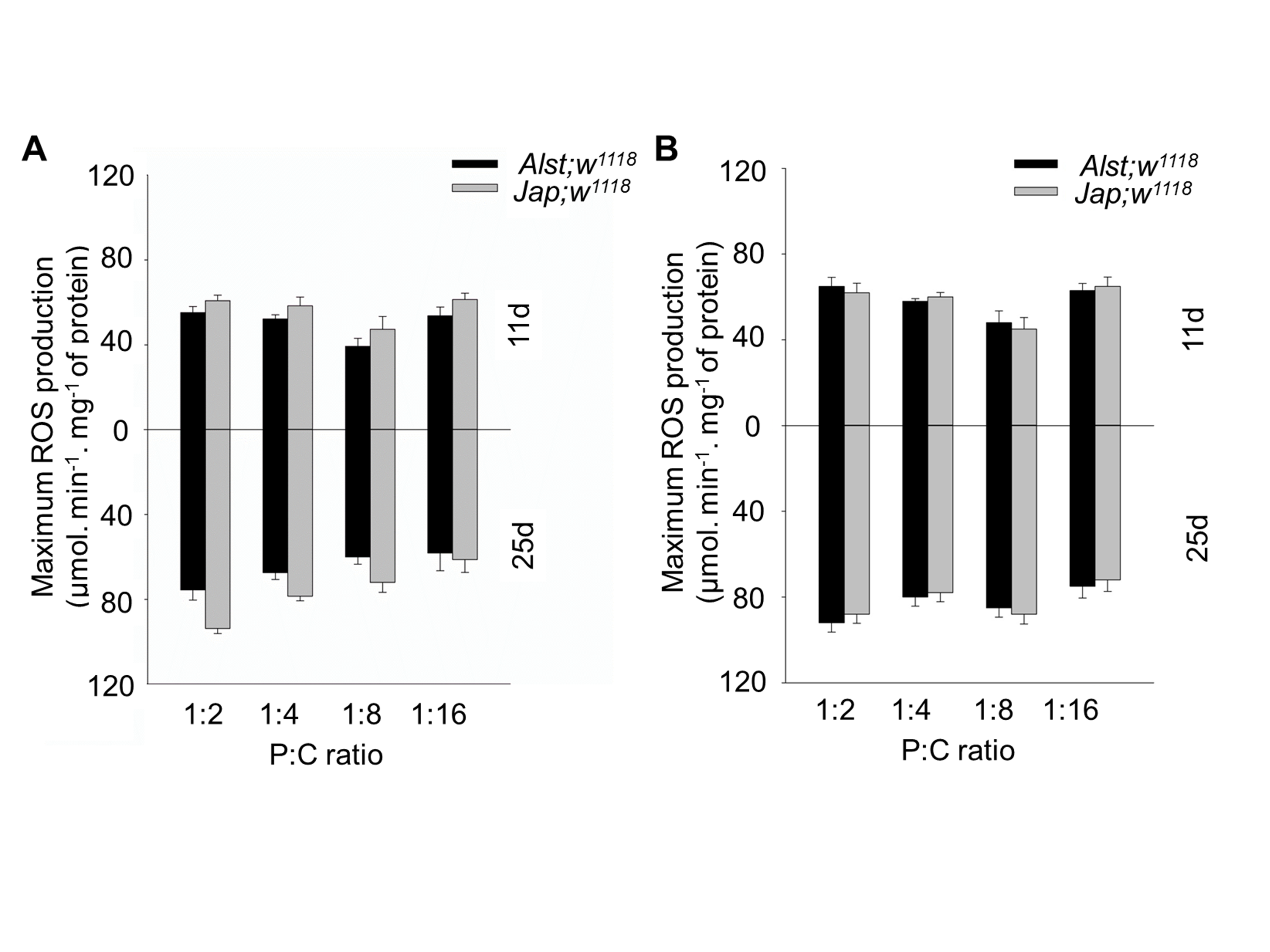

Supplement: S2 Fig — Flies were aged 11 (upper chart) and 25 d (lower chart). The protein: carbohydrate (P:C) diets were 1:2, 1:4, 1:8 and 1:16. (A) Males. (B) Females. Bar represents basal ROS production, and error bars show the standard error of the mean. (TIF) [file pone.0187554.s004.tif]

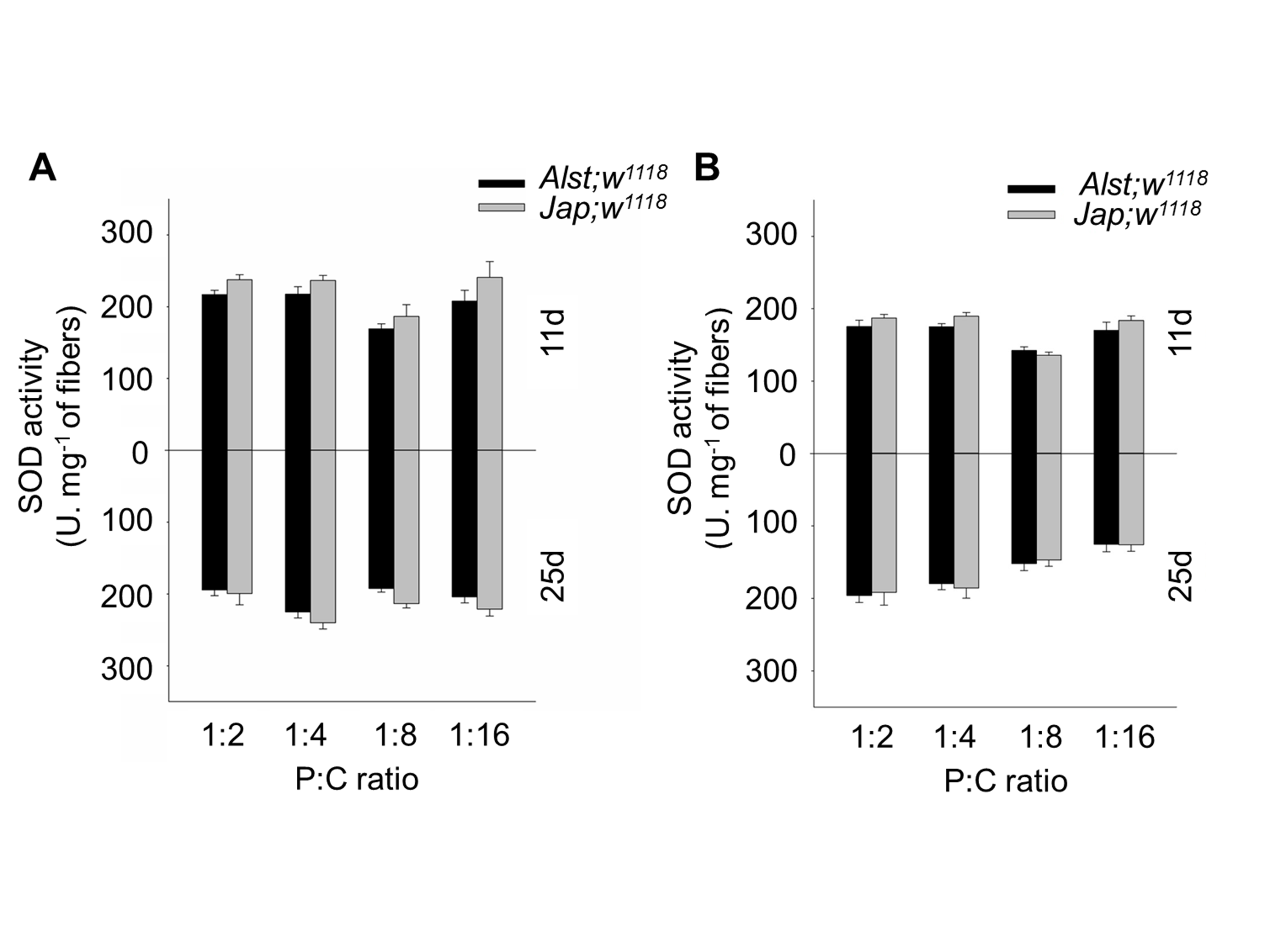

Supplement: S3 Fig — Flies were aged 11 (upper chart) and 25 d (lower chart). The protein: carbohydrate (P:C) diets were 1:2, 1:4, 1:8 and 1:16. (A) Males. (B) Females. Bar represents SOD activity, and error bars the standard error of the mean. (TIF) [file pone.0187554.s005.tif]

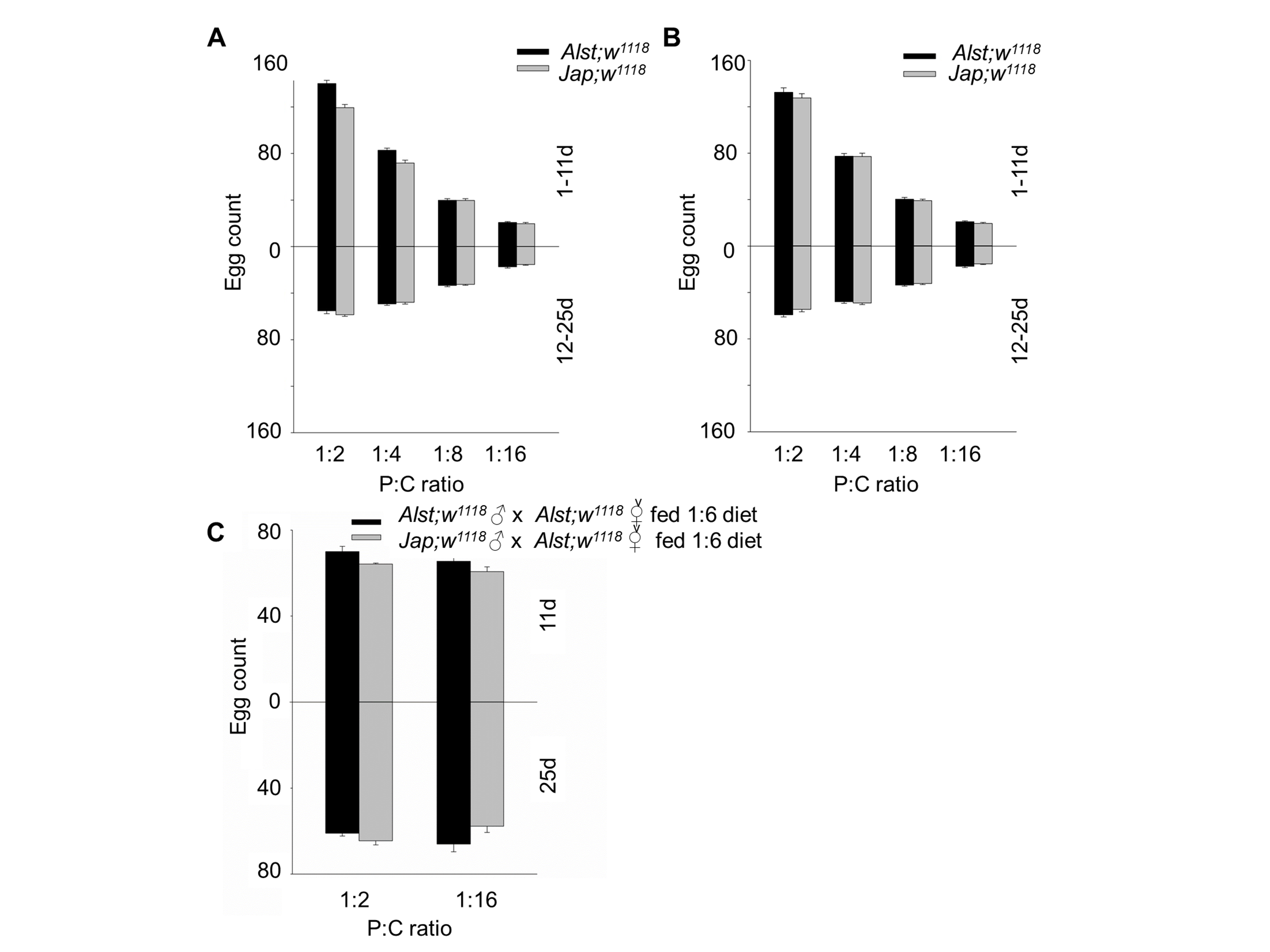

Supplement: S4 Fig — The flies were aged from 1–11 d (upper chart) and 12–25 d (lower chart). The protein: carbohydrate (P:C) diets were 1:2, 1:4, 1:8 and 1:16. (A) Males. (B) Females. (C) The fecundity of males (11 d or 25 d) mated with 5 d old virgin females fed on the intermediate 1:6 P:C diet. Bar represents total egg count, and error bars show standard error of the mean. (TIF) [file pone.0187554.s006.tif]

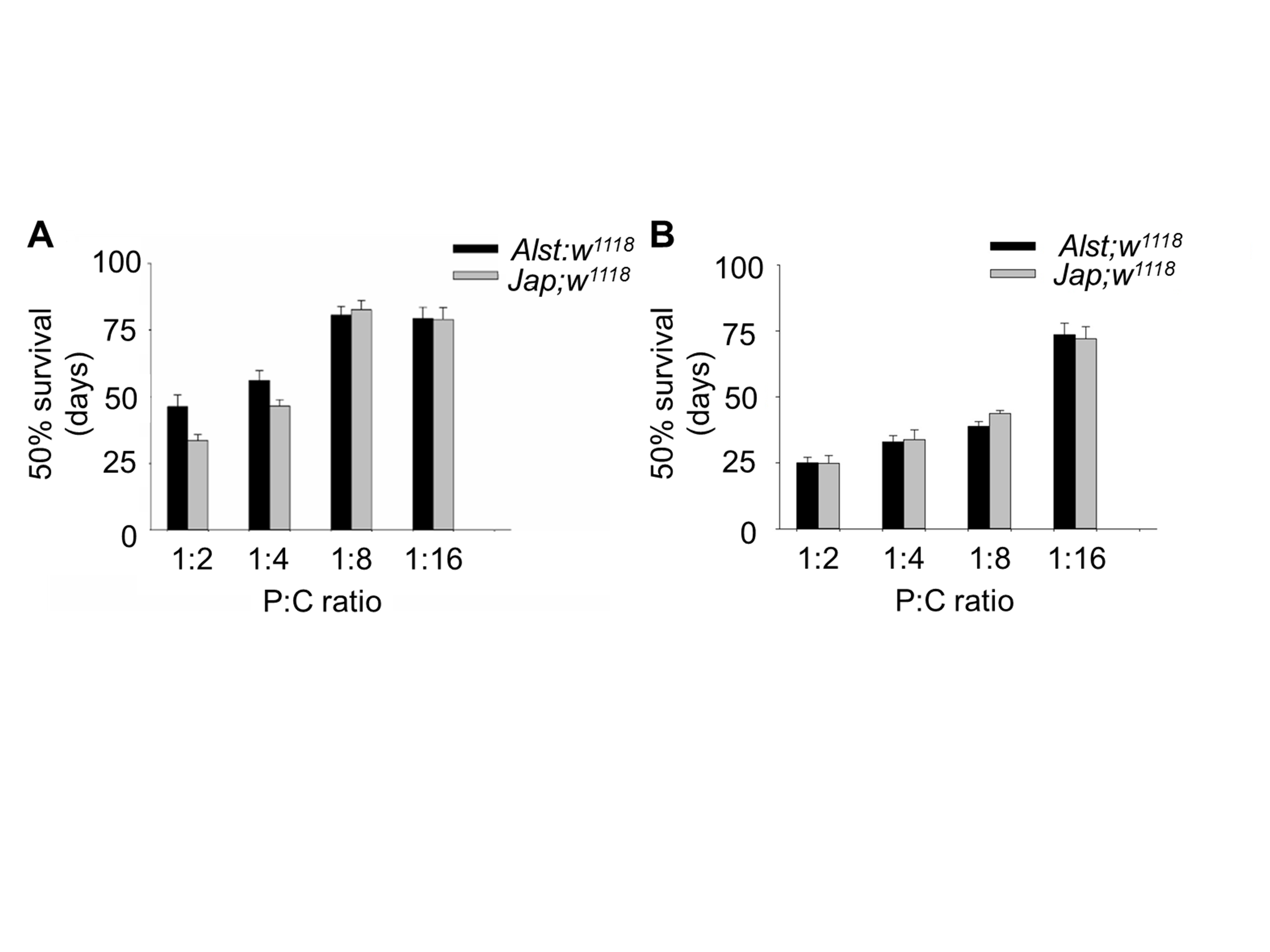

Supplement: S5 Fig — The protein: carbohydrate (P:C) diets were 1:2, 1:4, 1:8 and 1:16. (A) Males. (B) Females. Bar represents 50% survival, and error bars show standard error of the mean. (TIF) [file pone.0187554.s007.tif]

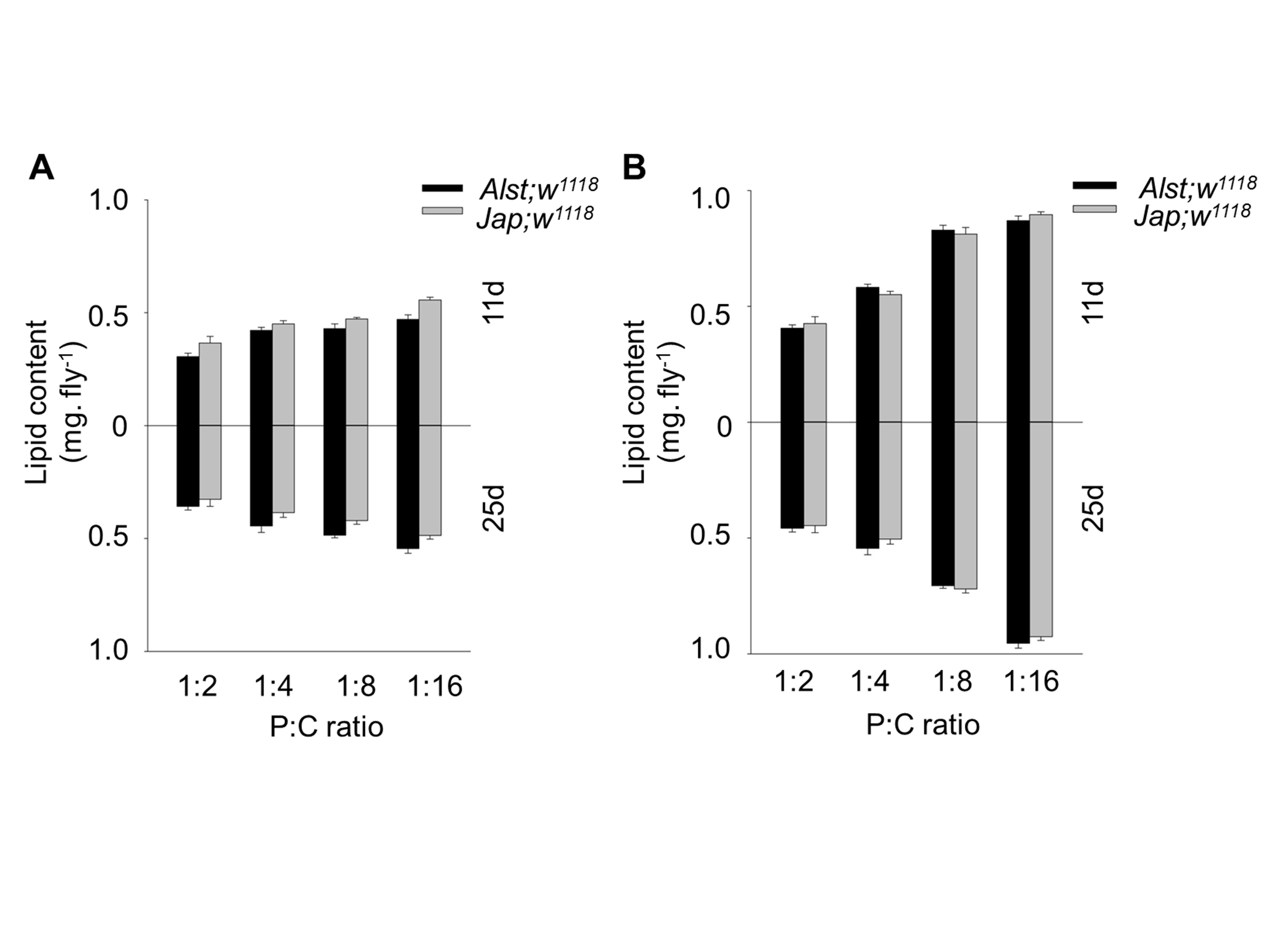

Supplement: S6 Fig — Flies were aged 11 d (upper chart) and 25 d (lower chart). The protein: carbohydrate (P:C) diets were 1:2, 1:4, 1:8 and 1:16. (A) Male. (B) Female. Bar represents lipid content, and error bars show standard error of the mean. (TIF) [file pone.0187554.s008.tif]
